# The Unconventional Cytoplasmic Sensing Mechanism for Ethanol Chemotaxis in Bacillus subtilis

**DOI:** 10.1128/mBio.02177-20

**Published:** 2020-10-06

**Authors:** Payman Tohidifar, Girija A. Bodhankar, Sichong Pei, C. Keith Cassidy, Hanna E. Walukiewicz, George W. Ordal, Phillip J. Stansfeld, Christopher V. Rao

**Affiliations:** a Department of Chemical and Biomolecular Engineering, University of Illinois at Urbana-Champaign, Urbana, Illinois, USA; b Department of Biochemistry, University of Illinois at Urbana-Champaign, Urbana, Illinois, USA; c Department of Biochemistry, University of Oxford, Oxford, United Kingdom; d School of Life Sciences, University of Warwick, Coventry, United Kingdom; e Department of Chemistry, University of Warwick, Coventry, United Kingdom; The Ohio State University

**Keywords:** chemotaxis, *Bacillus subtilis*, ethanol sensing, cytoplasmic sensing, chemoreceptor, molecular dynamics, NMR, signal transduction

## Abstract

Motile bacteria sense chemical gradients using chemoreceptors, which consist of distinct sensing and signaling domains. The general model is that the sensing domain binds the chemical and the signaling domain induces the tactic response. Here, we investigated the unconventional sensing mechanism for ethanol taxis in Bacillus subtilis. Ethanol and other short-chain alcohols are attractants for B. subtilis. Two chemoreceptors, McpB and HemAT, sense these alcohols. In the case of McpB, the signaling domain directly binds ethanol. We were further able to identify a single amino acid residue, Ala^431^, on the cytoplasmic signaling domain of McpB that, when mutated to serine, reduces taxis to alcohols. Molecular dynamics simulations suggest that the conversion of Ala^431^ to serine increases coiled-coil packing within the signaling domain, thereby reducing the ability of ethanol to bind between the helices of the signaling domain. In the case of HemAT, the myoglobin-like sensing domain binds ethanol, likely between the helices encapsulating the heme group. Aside from being sensed by an unconventional mechanism, ethanol also differs from many other chemoattractants because it is not metabolized by B. subtilis and is toxic. We propose that B. subtilis uses ethanol and other short-chain alcohols to locate prey, namely, alcohol-producing microorganisms.

## Abstract (precis)

Ethanol is a chemoattractant for Bacillus subtilis even though it is not metabolized and inhibits growth. B. subtilis likely uses ethanol to find ethanol-fermenting microorganisms to utilize as prey. Two chemoreceptors sense ethanol: HemAT and McpB. HemAT’s myoglobin-like sensing domain directly binds ethanol, but the heme group is not involved. McpB is a transmembrane receptor consisting of an extracellular sensing domain and a cytoplasmic signaling domain. While most attractants bind the extracellular sensing domain, we found that ethanol directly binds between intermonomer helices of the cytoplasmic signaling domain of McpB, using a mechanism akin to those identified in many mammalian ethanol-binding proteins. Our results indicate that the sensory repertoire of chemoreceptors extends beyond the sensing domain and can directly involve the signaling domain.

## INTRODUCTION

Many bacteria move in response to external chemical gradients through a process known as chemotaxis (1). Typically, bacteria migrate up gradients of chemicals that support their growth and down ones that inhibit it. These chemicals are commonly sensed using transmembrane chemoreceptors, which consist of an extracellular sensing domain and a cytoplasmic signaling domain along with a cytoplasmic HAMP domain that couples the other two domains. While a number of sensing mechanisms exist, the best-understood one involves the direct binding of the chemical to the extracellular sensing domain (2). In flagellated bacteria such as Bacillus subtilis and Escherichia coli, this binding event induces a conformational change in the cytoplasmic signaling domain that alters the autophosphorylation rate of an associated histidine kinase known as CheA (3). The phosphoryl group is then transferred to a soluble response regulator known as CheY, which modulates the swimming behavior of the bacterium by changing the direction of flagellar rotation. The chemical gradients themselves are sensed using a temporal mechanism involving sensory adaptation (4).

While many chemicals are sensed by the extracellular sensing domain, some are sensed by the cytoplasmic domains, typically using an indirect mechanism. For example, sugars transported by the phosphoenolpyruvate transfer system (PTS) are indirectly sensed through interactions between the PTS proteins and chemoreceptor signaling complexes (5, 6). In the case of E. coli, changes in intracellular pH are sensed by the cytoplasmic HAMP domain (7). The signaling domain of E. coli Tar is responsible for the attractant response to toluene and *o*-xylene (8). In addition, changes in osmolarity are sensed through alterations in the packing of the chemoreceptor cytoplasmic signaling domains (9). To our knowledge, however, there have been no reports of direct sensing by the chemoreceptor cytoplasmic signaling domain. This has not been particularly surprising given that the cytoplasmic signaling domain, which consists of a long dimeric four-helix coiled coil (10), lacks an obvious ligand-binding pocket.

In this work, we investigated chemotaxis to ethanol in B. subtilis. This short-chain alcohol is an attractant for B. subtilis even though it is not used as a carbon source and inhibits cell growth. Ethanol is directly sensed by two chemoreceptors, HemAT and McpB, inside the cytoplasm, where intracellular ethanol concentrations are similar to the extracellular concentrations due to the high permeability of the plasma membrane for short-chain aliphatic alcohols (11–16). Sensing by HemAT fits the conventional model where ethanol binds the cytoplasmic sensing domain. However, in the case of McpB, we found that ethanol is directly sensed by the cytoplasmic signaling domain using a mechanism analogous to those of many eukaryotic ethanol-binding proteins.

## RESULTS

### B. subtilis exhibits chemotaxis to short-chain alcohols.

**FIG 1:**
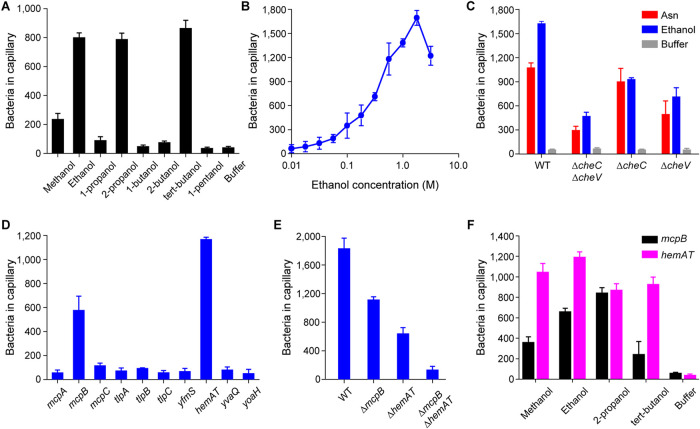
B. subtilis exhibits chemotaxis toward short-chain alcohols. (A) Responses of the wild-type strain to 0.5 M short-chain alcohols (in the capillaries) with increasing chain lengths (C_1_ to C_5_). (B) Dose-dependent responses of the wild-type (WT) strain to increasing concentrations of ethanol. (C) Responses of adaptation-deficient mutants to ethanol and asparagine. (D) Responses of mutants expressing single chemoreceptors to ethanol. (E) Responses of mutants lacking key chemoreceptors to ethanol. (F) Responses of mutants expressing McpB or HemAT as their sole chemoreceptor to short-chain alcohols. In these experiments, ethanol and asparagine concentrations within the capillaries were 1.78 M and 3.16 μM, respectively, unless otherwise mentioned. The negative-control responses of the strains expressing a single chemoreceptor to buffer were all <100 colonies per capillary. Error bars denote the standard deviations from three biological replicates performed on three separate days.

We employed the capillary assay to measure B. subtilis chemotaxis to alcohols with increasing chain lengths (C_1_ to C_5_). The resulting data show that B. subtilis exhibits chemotaxis to methanol, ethanol, 2-propanol, and *tert*-butanol. No significant responses to 1-propanol, 1-butanol, and 1-pentanol were observed (Fig. 1A). To elucidate the underlying sensing mechanism, we focused on ethanol because it is produced and utilized by a wide range of microorganisms in nature (17). We first measured the response to increasing ethanol concentrations using the capillary assay (Fig. 1B). Unlike many other attractants such as amino acids (18–20), a tactic response to ethanol was observed only at relatively high concentrations (>50 mM). The ethanol response peaked at 1.78 M (∼10% [vol/vol]). The response decreased at higher concentrations, most likely due to ethanol being toxic at these concentrations (21). It is important to note that the reported alcohol concentrations here are the initial alcohol levels inside the capillaries, and the concentrations sensed by the cells are much lower (see Discussion).

### All three adaptation systems contribute to ethanol taxis.

B. subtilis employs three adaptation systems, the methylation, CheC/CheD/CheYp, and CheV systems, for sensing chemical gradients (4, 22). To test whether these adaptation systems are involved in ethanol taxis, we employed mutants in which these systems were selectively inactivated. We first tested ethanol taxis using a mutant (Δ*cheC* Δ*cheV*) in which the CheC/CheD/CheYp and CheV adaptation systems were inactivated, leaving only the methylation system functional. Taxis to both ethanol and asparagine, which was used as a control, was reduced 30% in this mutant (Fig. 1C). We also observed reduced taxis in the Δ*cheC* and Δ*cheV* mutants, although the reduction was less than what was observed with the double mutant. Interestingly, the CheC/CheD/CheYp system appears to be more important for sensing ethanol gradients than for sensing asparagine gradients (Fig. 1C). We did not test a Δ*cheR* Δ*cheB* mutant, which lacks the two enzymes involved in the methylation system, because these mutants exhibit poor motility in general due to excessive tumbling.

### McpB and HemAT are the chemoreceptors for short-chain alcohols.

B. subtilis has 10 chemoreceptors (23). To determine the chemoreceptors involved in ethanol taxis, we first used the capillary assay to test mutants expressing just one chemoreceptor (19, 24, 25). Only strains expressing McpB or HemAT as their sole chemoreceptor were capable of ethanol taxis (Fig. 1D). The response was greater for strains expressing HemAT, suggesting that it is the main receptor for ethanol taxis. This is not surprising, as HemAT is more highly expressed than McpB (19,000 copies versus 6,200 copies) (23). We next tested the effect of deleting these chemoreceptors in the wild type. When either McpB or HemAT was deleted (Δ*mcpB* or Δ*hemAT*), we observed reduced taxis toward ethanol. The reduction was greater in the Δ*hemAT* mutant, again suggesting that HemAT is the main receptor for ethanol taxis. When both chemoreceptors were deleted in the wild type (Δ*mcpB* Δ*hemAT*), ethanol taxis was almost eliminated, whereas the mutant exhibited a normal response to proline, an amino acid attractant sensed by McpC (1,138.7 ± 34.6 cells versus 1,276.9 ± 54.1 cells for the wild type) (Fig. 1E). We also found that strains expressing McpB or HemAT as their sole chemoreceptor responded to methanol, 2-propanol, and *tert*-butanol (Fig. 1F). Strains expressing HemAT as their sole chemoreceptor exhibited stronger responses to these alcohols than strains expressing McpB alone, with the exception of 2-propanol, where the responses were similar.

### Chemotaxis to ethanol is independent of its metabolism.

**FIG 2:**
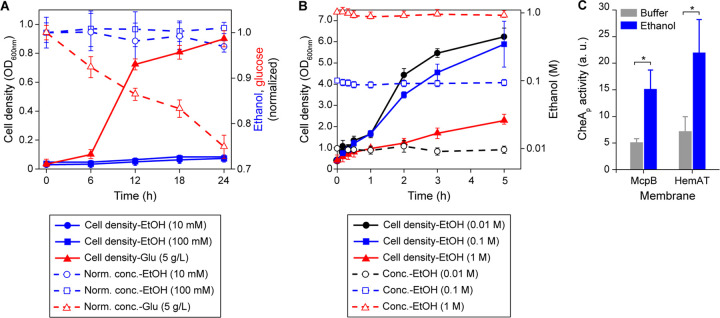
B. subtilis chemotaxis to ethanol is independent of its metabolism. (A) Cell growth in minimal medium supplemented with 10 mM ethanol (EtOH), 100 mM ethanol, and 5 g/liter glucose tested as a positive control. Dashed lines with the corresponding symbols depict normalized concentrations of the chemicals measured over the course of 24 h. (B) Cell growth in rich medium containing 10 mM ethanol, 100 mM ethanol, and 1 M ethanol. Dashed lines with open symbols depict absolute concentrations of ethanol measured under three different conditions over the course of 5 h. (C) Levels of phosphorylated CheA kinase complexed with CheW, CheD, and McpB or HemAT within the isolated membranes, in the presence of 1 M ethanol or buffer, as a negative control. Error bars denote the standard deviations from three biological replicates performed on three separate days. a.u., arbitrary units. *, *P < *0.05 (two-sided *t* test [correction for unequal variances was applied]).

Many bacteria metabolize ethanol (26). One possibility is that B. subtilis senses products of ethanol metabolism rather than ethanol itself. Indeed, such a mechanism occurs in Pseudomonas putida with regard to alcohol taxis (27). Therefore, we tested whether B. subtilis can grow on ethanol (Fig. 2A). These growth experiments were performed using the parental strain B. subtilis 168, which lacks the auxotrophies present in the chemotaxis strain OI1085. When cells were cultured in minimal medium with ethanol as the sole carbon source, no growth was observed. However, the cells grew when ethanol was replaced with glucose. We also tested B. subtilis 168 growth in rich medium containing different amounts of ethanol to determine whether the cells were able to consume ethanol even though it does not support growth as the sole carbon source. While the cells were able to grow in rich medium containing ethanol, no decreases in ethanol concentrations were observed (Fig. 2B). These results indicate that B. subtilis does not consume ethanol.

The oxidation of alcohols to aldehydes and subsequently to carboxylic acids can potentially change the redox state of the cells. This change could be perceived as a sensory signal through a process known as energy taxis (28). B. subtilis can ferment glucose to acetate and ethanol when grown in the presence of pyruvate or a mixture of amino acids (29, 30). In this process, alcohol dehydrogenase (ADH) reduces acetaldehyde to ethanol using NADH as the cofactor. Whether ADH can oxidize ethanol to acetaldehyde in B. subtilis is unknown. To test whether this occurs, we measured ADH activities using B. subtilis cell lysates prepared from aerobic and anaerobic cultures. As a positive control, ADH activities using E. coli cell lysates were also measured (31). No ADH activity was observed with B. subtilis lysates, whereas E. coli lysates obtained from anaerobic cultures had an ADH activity of 31.25 ± 1.85 U/ml. As expected, no ADH activity was detected with aerobic E. coli lysates. These results suggest that ethanol taxis in B. subtilis is independent of ethanol catabolism and is instead sensed directly by McpB and HemAT.

### Ethanol induces receptor-coupled kinase activity.

We next performed an *in vitro* receptor-coupled kinase assay to test whether ethanol is able to activate CheA kinase (32). This assay has been used to study how attractant binding to chemoreceptors modulates CheA kinase activity (22, 32). Briefly, membranes expressing either McpB or HemAT were isolated. The chemotaxis signaling proteins CheA, CheW, and CheD were then added to these membranes to final concentrations that matched their stoichiometry in wild-type cells. Using this assay, we found that ethanol activates CheA kinase in a dose-dependent manner with membranes containing either McpB or HemAT as the sole chemoreceptor. Ethanol concentrations as low as 10 mM were sufficient to activate CheA kinase in both cases (Fig. 2C; see also Fig. S1 in the supplemental material). These results indicate that ethanol can induce chemotaxis signaling *in vitro*. This assay, however, is unable to determine whether ethanol directly interacts with the chemoreceptors because the membranes might contain associated proteins that could be involved in signaling.

10.1128/mBio.02177-20.1 FIG S1 Ethanol induces receptor-coupled kinase activity. Levels of the phosphorylated CheA kinase protein complexed with CheW, CheD, and McpB (A) or HemAT (B) chemoreceptors within the isolated membranes or a receptorless membrane (negative control) (C) were measured in the presence of increasing ethanol concentrations. Asparagine at 3.16 μM was used as a positive control for membranes containing McpB, and buffer was used as a negative control in all experiments. Download FIG S1, TIF file, 1.0 MB. Copyright © 2020 Tohidifar et al. 2020 Tohidifar et al. This content is distributed under the terms of the Creative Commons Attribution 4.0 International license.

### The McpB cytoplasmic signaling domain is involved in ethanol sensing.

**FIG 3:**
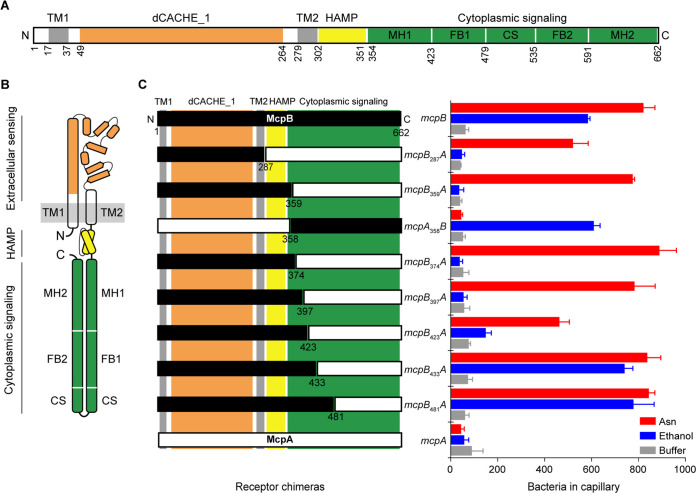
The McpB cytoplasmic signaling domain is involved in ethanol sensing. (A) Domain structures of McpB, McpA, TlpA, and TlpB. All four chemoreceptors consist of an extracellular sensing domain with a dCACHE_1 structure (orange) followed by transmembrane (TM1 and TM2) (gray), HAMP (yellow), and cytoplasmic signaling (green) domains. Three subdomains of the cytoplasmic signaling domain, classified as methylation (adaptation) helices (MH), the flexible (coupling) bundle (FB), and the conserved signaling (CS) (protein contact region) tip, are shown. (B) Cartoon structure of a monomer of the chemoreceptors. (C) Responses of mutants expressing chimeric receptors between McpA (white) and McpB (black) to 1.78 M ethanol, 3.16 μM asparagine, and buffer. Error bars denote the standard deviations from three biological replicates performed on three separate days.

We next investigated ethanol taxis using receptor chimeras involving McpB to provide further insight regarding the sensing mechanism (25, 33, 34). We focused on McpB due to its high amino acid similarity (57% to 65%) with three other B. subtilis chemoreceptors: McpA, TlpA, and TlpB. These four chemoreceptors all employ the same double Cache 1 sensing domain (2) and a highly conserved coiled-coil structure for their cytoplasmic signaling domain (10) (Fig. 3A and B). Unlike McpB, HemAT is not a transmembrane chemoreceptor. We attempted to construct chimeras involving HemAT, McpA, and YfmS, another soluble chemoreceptor. However, none were functional in the sense that they did not respond to ethanol or molecular oxygen, which is the conventional attractant for HemAT (24).

We created chimeras between McpB and McpA because the latter is not involved in ethanol taxis. In addition, we measured the response to asparagine because it is a known attractant for McpB, but not McpA, and it binds the extracellular sensing domain (18). We first fused the N-terminal region of McpB to the C-terminal region of McpA: *mcpB*_287_*A* and *mcpB*_359_*A*. We then tested whether strains expressing these chimeras as their sole chemoreceptor respond to ethanol using the capillary assay. Both mutants failed to respond to ethanol even though they still responded to asparagine (Fig. 3C). These results demonstrate that the extracellular sensing and cytoplasmic HAMP domains are not involved in sensing ethanol. Rather, the cytoplasmic signaling domain is involved. To verify our hypothesis, we tested an *mcpA*_358_*B* chimera. As expected, a strain expressing *mcpA*_358_*B* as its sole chemoreceptor responded to ethanol. This strain, however, does not respond to asparagine because it lacks the requisite McpB sensing domain (Fig. 3C).

Key features of chemoreceptor cytoplasmic signaling domains are the characteristic heptad repeats (labeled *a* to *g*) associated with their coiled-coil structure, where each repeat is equivalent to two helical turns. Based on sequence conservation and structural analyses of heptads from several bacterial and archaeal chemoreceptors, the cytoplasmic signaling domains are classified into three structurally distinct subdomains. These subdomains are known as the methylation (adaptation) helices, the flexible (coupling) bundle, and the conserved signaling tip (protein contact region) (10) (Fig. 3 and Fig. S2). To narrow down the region of these subdomains involved in ethanol sensing, we created *mcpB*_374_*A*, *mcpB*_397_*A*, *mcpB*_423_*A*, *mcpB*_433_*A*, and *mcpB*_481_*A* chimeras. Strains expressing *mcpB*_374_*A* and *mcpB*_397_*A* as their sole chemoreceptor did not respond to ethanol even though they still responded to asparagine. Strains expressing *mcpB*_423_*A* as their sole chemoreceptor exhibited a reduced response to ethanol and asparagine. However, when *mcpB*_433_*A* and *mcpB*_481_*A* were tested, the strains expressing these chimeras were able to respond to ethanol and asparagine at levels similar to those of the wild-type control (Fig. 3C). These results suggest that the region spanning residues 397 to 433 on McpB is involved in sensing ethanol. Furthermore, the region spanning residues 423 to 433 on McpB appears to be the principal region involved in ethanol sensing.

10.1128/mBio.02177-20.2 FIG S2 Amino acid sequences of three structural subdomains within the cytoplasmic signaling domains of B. subtilis transmembrane chemoreceptors. Amino acid sequences of three structural subdomains, known as the methylation helix (MH), the flexible bundle (FB), and the conserved signaling (CS) tip, within the cytoplasmic signaling domains of four B. subtilis transmembrane chemoreceptors are shown. For comparison, aligned amino acid sequences of the corresponding subdomains from four E. coli transmembrane chemoreceptors are also shown. Characteristic 7-residue repeats (heptads) along the helices are labeled *a* to *g*, and the corresponding amino acid sequences are separated by alternating gray and white colors. Download FIG S2, TIF file, 2.9 MB. Copyright © 2020 Tohidifar et al. 2020 Tohidifar et al. This content is distributed under the terms of the Creative Commons Attribution 4.0 International license.

### An McpB residue involved in ethanol sensing.

**FIG 4:**
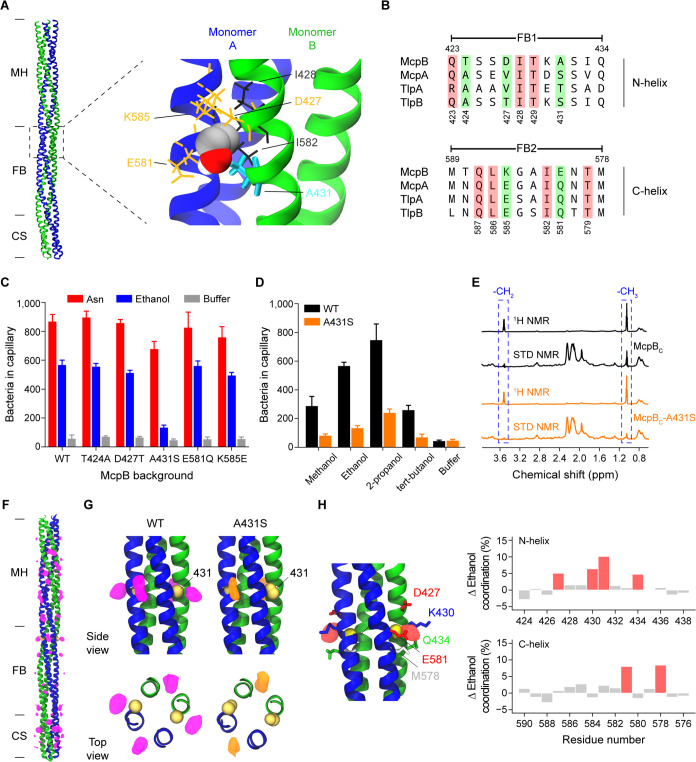
Alcohols are directly sensed by the cytoplasmic signaling domain of McpB. (A) Putative binding site within the primary ethanol-sensing region spanning residues 423 to 433 on the N-helix and the neighboring residues 579 to 589 on the C-helix of the McpB cytoplasmic signaling domain. (B) Amino acid sequence alignment of the primary ethanol-sensing region for McpB and the corresponding regions on McpA, TlpA, and TlpB. (C) Responses of strains expressing McpB mutants as their sole chemoreceptor to 1.78 M ethanol, 3.16 μM asparagine, and buffer. (D) Responses of strains expressing wild-type McpB and the McpB-A431S mutant as their sole chemoreceptor to 1.78 M short-chain alcohols and buffer. (E) ^1^H and STD-NMR spectra for 50 μM wild-type and mutant (A431S) recombinant McpB cytoplasmic regions (McpB_C_) spanning residues 305 to 662. Two peaks at 1.05 ppm and 3.51 ppm (shown inside dashed boxes) correspond to -CH_3_ and -CH_2_ epitopes of ethanol, respectively. (F) Density map of the average ethanol occupancy (purple) along the wild-type McpB cytoplasmic signaling domain (McpB_C_) spanning residues 352 to 662, as predicted by MD simulation. (G) Enlarged side and top views of the ethanol occupancy surrounding residue 431 (yellow) in wild-type (purple) and A431S mutant (orange) McpB_C_. (H) Difference map (red density) between wild-type and A431S mutant McpB_C_ surrounding residue 431 highlighting the loss of an intermonomer ethanol-binding site in the A431S mutant. Changes in protein-ethanol coordination highlight the putative amino acid residues (red bars) involved in ethanol binding. Error bars reported in panels C and D denote the standard deviations from three biological replicates performed on three separate days. Ethanol occupancy and coordination values were generated from three independent MD simulations.

The region spanning residues 397 to 433 on McpB is necessary for ethanol taxis. As a first step toward identifying the binding site, we performed *in silico* docking experiments with ethanol and the McpB dimer fragment spanning residues 390 to 435 on the N-helix and the neighboring residues 577 to 622 on the C-helix. The resulting data from the docking analysis yielded five distinct clusters of putative amino acid residues involving both the N-helix and the C-helix of the dimer fragment (Table S1 and Fig. S3A). We next aligned the amino acid sequences spanning residues 392 to 434 on the N-helix and the neighboring residues 578 to 620 on the C-helix of McpB, McpA, TlpA, and TlpB (Fig. S3B). Among the 20 putative binding residues, Thr^424^, Asp^427^, and Ala^431^ on the N-helix and Glu^581^ and Lys^585^ on the C-helix were not conserved between the four chemoreceptors and, thus, were targeted for mutational analysis (Fig. 4A and B). Mutants expressing McpB-T424A, McpB-D427T, McpB-E581Q, and McpB-K585E as their sole chemoreceptor exhibited responses to ethanol similar to those of wild-type *mcpB*. However, the strain expressing McpB-A431S as its sole chemoreceptor failed to respond to ethanol. In addition, all strains supported asparagine taxis, indicating that these mutated receptors were functional (Fig. 4C). We also measured the responses of the strain expressing McpB-A431S to methanol, 2-proponal, and *tert*-butanol in the capillary assay and observed reduced responses (Fig. 4D), suggesting that Ala^431^ is an important residue for alcohol taxis overall.

10.1128/mBio.02177-20.3 FIG S3 Identification of putative ethanol-binding sites on the cytoplasmic signaling domain of the McpB dimer. (A) Five different clusters of putative binding sites within the ethanol-sensing region spanning residues 390 to 435 on the N-helix and the neighboring residues 577 to 622 on the C-helix of the McpB dimer fragment, predicted by *in silico* docking experiments. Monomers A and B are shown in blue and green, respectively. (B) Amino acid sequence alignment of the ethanol-sensing region spanning residues 392 to 434 on the N-helix and the neighboring residues 578 to 620 on the C-helix of McpB and the corresponding regions on McpA, TlpA, and TlpB. Conserved and nonconserved putative ethanol-binding residues are highlighted in red and green, respectively. Download FIG S3, TIF file, 2.0 MB. Copyright © 2020 Tohidifar et al. 2020 Tohidifar et al. This content is distributed under the terms of the Creative Commons Attribution 4.0 International license.

10.1128/mBio.02177-20.8 TABLE S1 Putative ethanol-binding sites within the McpB ethanol-sensing region predicted by *in silico* docking experiments. Download Table S1, PDF file, 0.02 MB. Copyright © 2020 Tohidifar et al. 2020 Tohidifar et al. This content is distributed under the terms of the Creative Commons Attribution 4.0 International license.

### Ethanol directly binds to the McpB cytoplasmic signaling domain.

To test whether ethanol directly interacts with McpB, we conducted saturation transfer difference nuclear magnetic resonance (STD-NMR) experiments using recombinant McpB. STD-NMR has been used to measure weak interactions between proteins and their ligands (35–38). Briefly, in these experiments, the protein is selectively saturated at specific frequencies. The magnetization is then transferred to the surrounding, low-molecular-weight ligands in a distance-dependent manner. The ligand epitopes near the protein receive higher saturation (39), implying direct binding to the protein.

We first tested the McpB cytoplasmic region (McpB_C_) spanning residues 305 to 662, which corresponds to the HAMP and signaling domains (Fig. 3A). The resulting ^1^H spectrum for the McpB_C_ protein incubated with 3 mM ethanol (60-fold molar excess of the protein) is shown in Fig. 4E. Two peaks for ethanol appeared near 1.05 ppm and 3.51 ppm, which correspond to the -CH_3_ and -CH_2_ epitopes of ethanol, respectively. Ligand signals were also observed at the expected chemical shift values (1.05 ppm and 3.51 ppm) on the STD spectra. Additionally, the area under the STD peak corresponding to the -CH_2_ epitope was about 5-fold (18%) smaller than that of the -CH_3_ epitope (Fig. 4E), suggesting that the -CH_3_ moiety of ethanol is closer to the protein than its -CH_2_ moiety. Moreover, control experiments using 3 mM 1-pentanol, which is not an attractant, and McpB_C_ showed negligible STD peaks near the characteristic chemical shift values (3.5, 1.41, 1.18, and 0.8 ppm), suggesting that 1-pentanol does not bind McpB_C_ (Fig. S4A). As an additional negative control, we performed STD-NMR experiments using the McpA cytoplasmic region spanning residues 305 to 661 with 3 mM ethanol. Consistent with our *in vivo* results, we did not observe significant STD peaks near the characteristic chemical shift values (Fig. S4A). These results collectively indicate that ethanol directly interacts with the McpB cytoplasmic region.

10.1128/mBio.02177-20.4 FIG S4 Control experiments for *in vitro* binding measurements. (A, top) ^1^H and STD-NMR spectra for 50 μM the recombinant McpA cytoplasmic region (McpA_C_) spanning residues 304 to 661 with 3 mM ethanol (red). Two peaks at 1.05 ppm and 3.51 ppm (shown inside dashed boxes) correspond to the -CH_3_ and -CH_2_ epitopes of ethanol, respectively. (Bottom) ^1^H and STD-NMR spectra for 50 μM the recombinant McpB cytoplasmic region (McpB_C_) spanning residues 305 to 662 with 3 mM 1-pentanol (black). Peaks shown inside dashed boxes correspond to -CH epitopes of 1-pentanol as indicated by increasing numerical superscripts, with 1 corresponding to the first carbon adjacent to the hydroxyl group. (B) CD spectra of recombinant wild-type and mutant (A431S) McpB_C_ and recombinant McpA_C_, reported as mean residual ellipticity (MRE) values. (C) CD spectra of the recombinant HemAT signaling domain (HemAT_S_) spanning residues 177 to 432. Download FIG S4, TIF file, 0.5 MB. Copyright © 2020 Tohidifar et al. 2020 Tohidifar et al. This content is distributed under the terms of the Creative Commons Attribution 4.0 International license.

Strains expressing McpB-A431S as their sole chemoreceptor exhibited a reduced response to ethanol when tested in the capillary assay (Fig. 4C). To determine whether the A431S mutation reduces ethanol binding, we repeated the STD-NMR experiments with the recombinant McpB_C_-A431S protein. Because single mutations may impair the proper folding of proteins, we first measured the circular dichroism (CD) spectra for both the wild-type McpB_C_ and the McpB_C_-A431S proteins. We observed similar spectra for both proteins, which suggests that the mutant protein preserves the wild-type helical structure (Fig. S4B). We then performed STD-NMR experiments with McpB_C_-A431S in the presence of 3 mM ethanol. The resulting STD spectra showed reduced peaks near 1.05 ppm and 3.51 ppm compared to wild-type McpB_C_ (Fig. 4E). The saturation fractions of ethanol, which correspond to the ratios of the areas under the respective -CH_3_ peaks on STD and ^1^H spectra, are 0.23 for McpB_C_ and 0.1 for McpB_C_-A431S. These results imply that residue Ala^431^ has a role in ethanol binding to the McpB signaling domain.

### Molecular dynamics simulation suggests the A431S mutation reduces the affinity of ethanol for the McpB cytoplasmic signaling domain.

To gain insight regarding the ethanol-binding mechanism, we performed molecular dynamics (MD) simulations of the wild-type and A431S McpB cytoplasmic signaling dimers (residues 352 to 662) in the presence of ethanol. Our simulations demonstrate that ethanol can bind nonspecifically throughout the cytoplasmic signaling domain in both the wild-type and mutant McpB dimers, primarily interacting along the interhelical grooves of the four-helix bundle (Fig. 4F and Fig. S5A). A comparison of ethanol occupancies between wild-type and A431S mutant McpB showed little variation overall but exhibited a marked difference in the region immediately surrounding residue 431. In particular, while ethanol was observed to bind at both the inter- and intramonomer interfaces in the wild-type simulations, the intermonomer binding site associated with the residue 431 side chain was not present in the mutant simulations (Fig. 4G), suggesting that the A431S mutation reduces the binding affinity of ethanol. Indeed, within the flexible-bundle region, the residues displaying the greatest change in ethanol coordination between the wild type and the A431S mutant form a concentric pocket centered on residue 431 at the intermonomer interface (Fig. 4H).

10.1128/mBio.02177-20.5 FIG S5 Ethanol and knob residue occupancy along the McpB coiled coil. (A) Density maps of the average ethanol occupancy along the wild-type (purple) and the A431S mutant (orange) McpB cytoplasmic signaling domains (McpB_C_). Differences between the wild type and the A431S mutant (red density) reveal three distinct putative ethanol-binding sites (S1, S2, and S3). Average changes in protein-ethanol coordination highlight the putative amino acid residues (red bars) involved in ethanol binding in each site. (B) Distribution of knob residues (purple, space filling) on the McpB cytoplasmic signaling dimer as identified using SOCKET (hole residues are not shown). The close-up depicts the identified knobs near residue Ala^431^ (yellow). In addition to Ala^431^, residues Ala^583^ and Lys^585^ (cyan) are predicted to have higher average knob occupancies in McpB_C_-A431S than in wild-type McpB_C_. Data and error bars associated with the knob occupancies in panel B denote the means ± standard deviations from three independent simulations. Download FIG S5, TIF file, 2.4 MB. Copyright © 2020 Tohidifar et al. 2020 Tohidifar et al. This content is distributed under the terms of the Creative Commons Attribution 4.0 International license.

Our analyses identified another interesting feature of ethanol binding, namely, that it is able to penetrate the surface of the McpB cytoplasmic domain to bind within the core of the coiled coil. In particular, we observed that ethanol entered between the individual helices of the four-helix bundle at two locations in the methylation-helix region: one involving N-helix residues 393 to 400 and C-helix residues 613 to 617 and the other involving N-helix residues 382 to 387 and C-helix residues 628 to 631 (Fig. S5A). While ethanol binding to these regions was observed in both the wild-type and A431S mutant simulations, the wild-type binding events resulted in longer dwell times, giving rise to the difference in ethanol coordination observed in these regions (Fig. S5A). Preliminary analysis of the two sites, however, suggests that they do not themselves play a significant role in signaling. The latter is located outside the region involved in ethanol sensing (Fig. 3C), and the former, except for residue Glu^399^, is highly conserved among the four chemoreceptors (Fig. S3B). Indeed, we did not observe a significant reduction in response to ethanol compared to the wild-type control when we tested a mutant expressing McpB-E399K as its sole chemoreceptor in the capillary assay (569 ± 29.1 cells versus 586.1 ± 9.0 cells, respectively). Nevertheless, these observations hint at a signaling mechanism in which ethanol may penetrate to the core of the cytoplasmic domain, where it can affect the packing and overall stability of the bundle.

To investigate the above-described packing hypothesis further, we analyzed the strength of knobs-in-holes interactions in the region surrounding residue 431 over the course of the simulations. We observed that the A431S mutation leads to an increased occupancy of the residue 431 knob itself as well as nearby knobs on the C-helix at positions 583 and 585 (Fig. S5B), indicating stronger hydrophobic interactions between the individual helices. Therefore, our simulation results suggest that the A431S substitution, which decreases the McpB ethanol response, not only reduces the direct binding of ethanol but also strengthens coiled-coil packing in the region. One possibility is that the reduced local concentration of ethanol and improved packing in the A431S mutant decrease the ability of ethanol to intercalate with the knobs-into-holes interactions near residue 431 and, thus, its ability to induce signaling.

### The HemAT sensing domain helices are directly involved in ethanol sensing.

**FIG 5:**
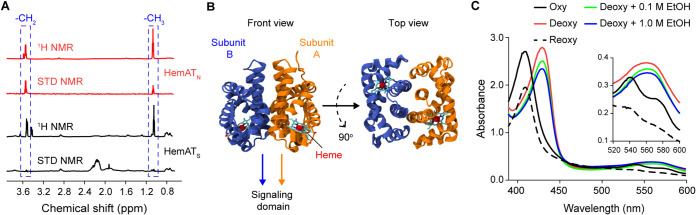
Ethanol directly binds within the helices of the HemAT sensing domain. (A) ^1^H and STD-NMR spectra for 50 μM the recombinant HemAT C-terminal signaling domain (HemAT_S_) spanning residues 177 to 432 and for 50 μM the recombinant HemAT N-terminal sensing domain (HemAT_N_) spanning residues 1 to 178, in the presence of 3 mM ethanol. Two peaks at 1.05 ppm and 3.51 ppm (shown inside dashed boxes) correspond to -CH_3_ and -CH_2_ epitopes of ethanol, respectively. (B) Crystal structure of the dimeric HemAT sensing domain. (C) UV spectra of recombinant HemAT_N_ in the absence and presence of molecular oxygen, 0.1 M ethanol, and 1.0 M ethanol.

HemAT is a cytoplasmic chemoreceptor that consists of an N-terminal sensing domain and a C-terminal signaling domain. To determine whether the HemAT signaling domain is also involved in ethanol sensing, we conducted STD-NMR experiments with the purified signaling domain (HemAT_S_), spanning residues 177 to 432, and the purified sensing domain (HemAT_N_), spanning residues 1 to 178, of HemAT in the presence of 3 mM ethanol. The STD spectra with the HemAT signaling domain showed negligible peaks near the expected chemical shift values (1.05 ppm and 3.51 ppm), while the resulting ^1^H and STD spectra with HemAT_N_ showed clear peaks near 1.05 ppm and 3.51 ppm, which correspond to the -CH_3_ and the -CH_2_ moieties of ethanol. The ratios of the areas in the STD spectra to those in the ^1^H spectra were 0.27 for the -CH_3_ moiety and 0.85 for the -CH_2_ moiety, suggesting that the -CH_2_ moiety of ethanol is closer to the protein than its -CH_3_ moiety (Fig. 5A). These results collectively indicate that ethanol binds the sensing domain of HemAT.

The sensing domain of the HemAT dimer is composed of a four-helical bundle as its core and a heme group in each subunit (Fig. 5B), which is known to bind molecular oxygen (40). UV spectral analyses have shown that the oxygen molecule binds the heme group by forming hydrogen bonds with 6-coordinate ferrous heme (41, 42). To determine whether the heme group also interacts with ethanol, we conducted UV spectroscopy experiments with the purified HemAT sensing domain (HemAT_N_) and ethanol. As a control, we first measured the UV absorption of both oxygenated and deoxygenated forms of the protein to verify that the heme group on the purified protein is functional. Consistent with previous reports (24, 41, 43), the oxygenated form of HemAT_N_ exhibited three major canonical peaks at 412 nm (Soret), 544 nm (β-band), and 578 nm (α-band), and the dithionite-reduced deoxygenated form of the protein exhibited two major peaks at 434 nm and 556 nm (Fig. 5C). Next, we measured the UV absorption of the deoxygenated HemAT sensing domain in the presence of various concentrations of ethanol. The resulting spectra showed two major peaks at 434 nm and 556 nm, similar to what we observed with the deoxygenated form of the protein in the absence of any ligand (Fig. 5C). These results imply that the heme group does not interact with ethanol. Rather, they suggest that ethanol binds to the alpha helices of the HemAT sensing domain. It is noteworthy that while ethanol binds to the alpha helices in both McpB and HemAT, the binding mode appears to be different. The STD-NMR experiments suggest that the -CH_3_ moiety of ethanol is closer to McpB than its -CH_2_ moiety, whereas the ethanol binding direction is reversed in the case of HemAT.

## DISCUSSION

We found that B. subtilis performs chemotaxis to multiple short-chain alcohols. These alcohols are directly sensed by two chemoreceptors, McpB and HemAT. McpB is a transmembrane chemoreceptor with an extracellular sensing domain and a cytoplasmic signaling domain, which are linked by a cytoplasmic HAMP domain. It is known to sense the amino acid asparagine and alkaline environments as attractants using the extracellular sensing domain (18, 25). HemAT, on the other hand, is a soluble chemoreceptor that consists of a sensing and a signaling domain but lacks a HAMP domain. Its myoglobin-like sensing domain contains heme and is known to bind molecular oxygen (24, 42). Using chimeric receptors and STD-NMR, we found that short-chain alcohols are directly sensed by the cytoplasmic signaling domain of McpB and the sensing domain of HemAT. In the case of HemAT, the alcohols do not appear to bind heme; rather, they likely bind between the helices encapsulating the heme.

Among the alcohols tested, ethanol is the most likely physiological attractant because it is produced by many microorganisms and is prevalent in nature (17). As a consequence, we focused on this chemical. Curiously, ethanol is not consumed by B. subtilis, suggesting that it is used for purposes other than nutrition. One possibility is that B. subtilis uses ethanol to locate prey, which could explain why B. subtilis is attracted to a chemical that nominally inhibits its growth. The most likely prey are Crabtree-positive yeast such as Saccharomyces cerevisiae, which produces ethanol at high concentrations even during aerobic growth (44). Indeed, B. subtilis can lyse S. cerevisiae cells through the production of cell wall-degrading compounds (see Fig. S6 in the supplemental material) (45–47).

10.1128/mBio.02177-20.6 FIG S6 Antifungal activity of B. subtilis strains. (A) Growth inhibition of S. cerevisiae by supernatants from cell cultures of the B. subtilis OI1085 laboratory chemotaxis strain and the undomesticated NCBI 3610 strain grown overnight was measured using a disc diffusion assay. Similar experiments were conducted using only water or LB broth, instead of the culture supernatant, as negative controls. (B) Chemotaxis responses of the B. subtilis OI1085 laboratory chemotaxis strain and the undomesticated NCBI 3610 strain to 1.78 M ethanol and buffer. Data and error bars shown in panel B denote the means ± standard deviations from three biological replicates performed on three separate days. Download FIG S6, TIF file, 1.3 MB. Copyright © 2020 Tohidifar et al. 2020 Tohidifar et al. This content is distributed under the terms of the Creative Commons Attribution 4.0 International license.

Aside from ethanol, methanol may also be a physiological attractant. It is a by-product of pectin degradation and, as a consequence, can contaminate alcoholic beverages (48). However, B. subtilis does not consume methanol because it lacks methanol dehydrogenase activity (49). Similar to ethanol, B. subtilis may use methanol to locate prey, in this case pectin-degrading microorganisms.

These speculations are in line with the results from a previous study where no correlation was observed between the metabolic and chemotactic preferences of B. subtilis for amino acids (50). That study proposed that B. subtilis uses amino acid gradients as cues to locate sources of nutrients, for example, during plant root colonization (50, 51). The only other bacterium known to exhibit taxis toward alcohols is Pseudomonas putida (27). However, this bacterium consumes alcohols. In addition, it does not directly sense these alcohols but rather senses the by-products of their degradation, namely, carboxylic acid. Finally, alcohol taxis is also observed in E. coli and Ralstonia pseudosolanacearum. In these bacteria, however, alcohols are sensed as repellents (52, 53).

A key difference between taxis to alcohols and conventional attractants such as amino acids is their respective sensitivities. Amino acids are sensed at micromolar concentrations, whereas alcohols are sensed at millimolar concentrations. However, it should be noted that while low millimolar concentrations (e.g., 3 mM) of ethanol can bind McpB and HemAT *in vitro*, much higher ethanol levels are required for optimal chemotaxis in the capillary assay experiments. The disparity may be due to the architecture of the capillary assay. Briefly, chemotaxis to ethanol occurs when ethanol concentrations in the capillaries are as low as 50 mM (190.1 ± 44.5 cells versus 42.3 ± 6.2 cells for buffer), and the response to ethanol peaks when capillaries contain about 2 M ethanol. These concentrations, however, do not reflect the ethanol concentrations that cells experience near the mouth of the capillary in the pond. Three-dimensional simulations based on finite-element analysis of ethanol diffusion from a capillary into a pond indicate that the ethanol concentration falls dramatically, 10 to 50 times, compared with the initial ethanol concentration in the capillary near the mouth of the capillary (distances of <0.5 mm) (Fig. S7). These simulation results suggest that cells are able to respond to ethanol levels ranging from 1 to 200 mM. That said, the weak affinity for alcohols is not surprising because most ethanol receptors in mammals also exhibit a weak affinity for ethanol (54).

10.1128/mBio.02177-20.7 FIG S7 Simulation of ethanol diffusion in the capillary assay. (A) The three-dimensional finite-element computation domain consists of the ethanol column in the capillary and the region near the mouth of the capillary in the pond. (B) Normalized ethanol concentration profile along the center line of the capillary and the pond at three different time points. (C) Normalized ethanol concentration dynamics near the mouth of the capillary in the pond during a 30-min-long assay. In panels B and C, the ethanol concentration is normalized to the initial ethanol concentration in the capillary. The initial ethanol level in the pond was set to zero. Download FIG S7, TIF file, 0.6 MB. Copyright © 2020 Tohidifar et al. 2020 Tohidifar et al. This content is distributed under the terms of the Creative Commons Attribution 4.0 International license.

The question, then, is whether ethanol is actually an attractant for B. subtilis given that relatively high concentrations are necessary to elicit taxis. Overripe fruits provide one potential source for high ethanol concentrations, where concentrations can exceed 1 M (55). In addition, flooded plant roots can provide another source at millimolar concentrations (56–58). In this case, B. subtilis perhaps uses ethanol to locate roots for colonization to initiate symbiosis (51). These observations suggest that ethanol taxis can indeed occur in the environment. Whether the other alcohols reach such concentrations in the environment is not known.

Perhaps the most interesting aspect of ethanol taxis involves the sensing mechanism. Typically, small-molecule attractants bind the extracellular sensing domain. The main exceptions are PTS sugars, which are sensed indirectly through the PTS system (5, 6). Ethanol is sensed intracellularly. In the case of HemAT, this distinction is minor, as ethanol binds the sensing domain albeit one normally associated with oxygen sensing. In the case of McpB, the cytoplasmic signaling domain is involved in sensing ethanol through direct binding. This appears to be the first documented case of the cytoplasmic signaling domain being directly involved in sensing. While we were able to establish that ethanol binds the McpB cytoplasmic signaling domain using genetics and STD-NMR, the details of the binding and signaling mechanisms are still somewhat opaque. In particular, it is not clear whether ethanol exerts its effect precisely at residue 431 or possibly at one or multiple other positions along the lengthy cytoplasmic domain. Molecular dynamics simulations suggest that ethanol can bind nonspecifically at several places on the McpB cytoplasmic surface as well as penetrate to the core of the four-helix bundle, at least within the methylation helix region. Although we did not observe ethanol entering the bundle core near residue 431 in our simulations, it may do so on longer time scales or in particular signaling states. In addition, precise molecular details of how ethanol binding induces signaling in wild-type McpB remain to be worked out. The enhanced packing interactions in McpB-A431S, which does not respond to ethanol, suggest that A431 may disrupt or loosen packing, leading to changes in the overall stability of McpB that can be transmitted to the kinase. This idea is in line with numerous previous studies of the E. coli Tsr and Tar chemoreceptors, for example, that suggest that changes in periplasmic ligand binding and the adaptation state affect packing throughout the cytoplasmic bundle (5, 6).

Many aspects of ethanol sensing in B. subtilis are analogous to mechanisms observed in higher eukaryotes. Alcohols generally bind to proteins with low affinities, and relatively high concentrations of alcohols are required to induce behavioral effects. For example, ligand-gated ion channel receptors such as the *N*-methyl-d-aspartate (NMDA)-type glutamate receptors, γ-aminobutyric acid type A (GABA_A_) receptors, and glycine receptors all exhibit weak affinities for ethanol (>10 mM) (54). Although the binding sites on these proteins are not well characterized, ethanol is thought to bind helical regions in most cases. In the case of the GABA_A_ receptor, for example, ethanol binds within a small cavity between two transmembrane helices (TM2 and TM3) (59). Molecular dynamics studies show that ethanol modulates the receptor states by stabilizing helical crossing angles with a “wringing motion” (60, 61). Ethanol inhibition of the NMDA receptor is regulated by counteracting forces on M3 helices of the receptors with additional interactions with side chains (62). Potassium channels are also affected by >100 mM ethanol concentrations. Kinetic and structural studies of Shaw2 K^+^ channels have shown that the alpha-helical propensity of the loop in the pore-forming subunit is important for ethanol binding (63). Similarly, in the case of the odorant-binding protein LUSH from Drosophila melanogaster, a small cavity between the alpha helices accommodates a single ethanol molecule, whose hydroxyl group forms hydrogen bonds with neighboring threonine (Thr^57^) and serine (Ser^52^) residues (64). The binding motif found in LUSH is shared by the GABA_A_ receptor, the glycine receptor, and the *Drosophila* Shaw2 K^+^ channel (64), suggesting a common alcohol-binding mechanism in eukaryotes.

Experimental and computational studies of the ion channel GLIC in the bacterium Gloeobacter violaceus also point to a mechanism of alcohol binding within cavities between transmembrane helices (65). Analysis of binding sites from structural studies suggests that ethanol preferentially binds helices with amphipathic surfaces (54, 66, 67). The sensing mechanisms for these proteins typically involve the replacement of water molecules with ethanol within small hydrophobic cavities between two or more helices. Indeed, an analogous mechanism appears to be employed by the B. subtilis chemoreceptors. Given the reported similarities in the modes of action of ethanol in both prokaryotic and eukaryotic proteins, the model hypothesized in this investigation could provide evolutionary clues on the mechanisms of alcohol-sensing proteins.

## MATERIALS AND METHODS

### Chemicals and growth media.

The following media were used for cell growth: Luria-Bertani (LB) broth (1% tryptone, 0.5% yeast extract, and 0.5% NaCl), tryptone broth (TB) (1% tryptone and 0.5% NaCl), tryptose blood agar base (TBAB) (1% tryptone, 0.3% beef extract, 0.5% NaCl, and 1.5% agar), yeast-peptone-dextrose (YPD) broth (1% yeast extract, 2% peptone, and 2% dextrose), and capillary assay minimal medium (CAMM) [50 mM potassium phosphate buffer (pH 7.0), 1.2 mM MgCl_2_, 0.14 mM CaCl_2_, 1 mM (NH_4_)_2_SO_4_, 0.01 mM MnCl_2_, and 42 μM ferric citrate]. Chemotaxis buffer consists of 10 mM potassium phosphate buffer (pH 7.0), 0.14 mM CaCl_2_, 0.3 mM (NH_4_)_2_SO_4_, 0.1 mM EDTA, 5 mM sodium lactate, and 0.05% (vol/vol) glycerol. All alcohols used in this study were purchased from Fisher Scientific, Inc.

### Strains and plasmids.

All strains and plasmids used in this work are listed in Tables 1 and 2, respectively. Chemotaxis experiments were performed with derivatives of B. subtilis OI1085. Growth experiments were performed using B. subtilis 168, which is the parental strain. The undomesticated B. subtilis strain NCBI 3610 and the Saccharomyces cerevisiae CEN.PK113-7D yeast strain were used in the antimicrobial diffusion assays. All cloning was performed using NEB 5-alpha competent E. coli (New England BioLabs). All oligonucleotides used in this study are provided in Table S2 in the supplemental material.

**TABLE 1:** Strains used in this study

Strain	Relevant genotype or description	Source or reference
5-alpha	E. coli cloning host	New England BioLabs
BL21(DE3)	E. coli protease-deficient expression host	Novagen
GBS111	Saccharomyces cerevisiae CEN.PK113-7D	
NCBI3610	Undomesticated wild-type B. subtilis isolate	
OI3269	Bacillus subtilis 168 *trpC2*	
OI1085	*trpF7 hisH2 metC133 che^+^*	97
PTS375	Δ*cheC* Δ*cheV*	This work
PTS097	Δ*cheC*	25
PTS135	Δ*cheV*	25
PTS185	Δ*mcpB*	25
PTS328	Δ*hemAT*	This work
PTS238	Δ*mcpB* Δ*hemAT*	This work
OI3545	Δ10*mcp* Erm^r^ Cm^r^ Kan^r^ *che*^+^	24
OI3921	OI3545 *amyE5720*::*mcpA* Spc^r^	98
OI3605	OI3545 *amyE5720*::*mcpB* Spc^r^	5
OI3974	OI3545 *amyE5720*::*mcpC* Spc^r^	5
OI4474	OI3545 *amyE5720*::*tlpA* Spc^r^	25
OI4475	OI3545 *amyE5720*::*tlpB* Spc^r^	25
OI4483	OI3545 *amyE5720*::*tlpC* Spc^r^	25
OI4476	OI3545 *amyE5720*::*yfmS* Spc^r^	25
OI4477	OI3545 *amyE5720*::*yvaQ* Spc^r^	25
OI4482	OI3545 *amyE5720*::*hemAT* Spc^r^	25
OI4479	OI3545 *amyE5720*::*yoaH* Spc^r^	25
PTS522	OI3545 *amyE5720*::*mcpB*[*M1-V287*] *mcpA*[*L287-E661*]	This work
PTS529	OI3545 *amyE5720*::*mcpB*[*M1-Q359*] *mcpA*[*D359-E661*]	This work
GBS103	OI3545 *amyE5720*::*mcpB*[*M1-A374*] *mcpA*[*S374-E661*]	This work
GBS104	OI3545 *amyE5720*::*mcpB*[*M1-N397*] *mcpA*[*E397-E661*]	This work
GBS142	OI3545 *amyE5720*:: *mcpB*[*M1-Q423*] *mcpA*[*A423-E661*]	This work
GBS090	OI3545 *amyE5720*::*mcpB*[*M1-I433*] *mcpA*[*Q433-E661*]	This work
GBS091	OI3545 *amyE5720*::*mcpB*[*M1-I481*] *mcpA*[*Q433-E661*]	This work
PTS252	OI3545 *amyE5720*::*mcpA*[*M1-Q358*] *mcpB*[*D359-E662*]	This work
GBS149	OI3545 *amyE5720*::*mcpB*[*A431S*]	This work
GBS176	OI3545 *amyE5720*::*mcpB*[*T424A*]	This work
GBS175	OI3545 *amyE5720*::*mcpB*[*D427T*]	This work
GBS158	OI3545 *amyE5720*::*mcpB*[*E581Q*]	This work
GBS170	OI3545 *amyE5720*::*mcpB*[*K585E*]	This work
GBS192	OI3545 *amyE5720*::*mcpB*[*E399K*]	This work

**TABLE 2:** Plasmids used in this study

Plasmid	Description	Source or reference
pET28a(+)	His-tagged cloning vector for protein purification; Kan^r^	Novagen
pJSpe	Modified pJOE8999 optimized for Gibson assembly of homology templates; Amp^r^ Kan^r^	25
pPT037	pJSpe::*cheV* (for *cheV* knockout)	25
pPT058	pJSpe::*mcpB* (for *mcpB* knockout)	25
pPT053	pJSpe::*hemAT* (for *hemAT* knockout)	This work
pAIN750	B. subtilis empty vector for integration at *amyE*; Amp^r^ Spc^r^	98
pPT200	pAIN750::*mcpB*[*M1-V287*] *mcpA*[*L287-E661*]	This work
pPT205	pAIN750::*mcpB*[*M1-Q359*] *mcpA*[*D359-E661*]	This work
pGB42	pAIN750::*mcpB*[*M1-A374*] *mcpA*[*S374-E661*]	This work
pGB43	pAIN750::*mcpB*[*M1-N397*] *mcpA*[*E397-E661*]	This work
pGB34	pAIN750::*mcpB*[*M1-I433*] *mcpA*[*Q433-E661*]	This work
pGB64	pAIN750::*mcpB*[*M1-Q423*] *mcpA*[*A423-E661*]	This work
pGB35	pAIN750::*mcpB*[*M1-L481*] *mcpA*[*R481-E661*]	This work
pPT086	pAIN750::*mcpA*[*M1-Q358*]*-mcpB*[*D359-E662*]	This work
pGB65	pAIN750::*mcpB*[*A431S*]	This work
pGB83	pAIN750::*mcpB*[*T424A*]	This work
pGB82	pAIN750::*mcpB*[*D427T*]	This work
pGB67	pAIN750::*mcpB*[*E581Q*]	This work
pGB79	pAIN750::*mcpB*[*K585E*]	This work
pGB94	pAIN750::*mcpB*[*E399K*]	This work
pPT262	His_6_-C-terminal McpB expression plasmid, pET28(a)::*mcpB_C_*	This work
pGB78	His_6_-C-terminal McpB_C_-A431S expression plasmid, pET28(a)::*mcpB_C_*[*A431S*]	This work
pGB53	His_6_-C-terminal McpA expression plasmid, pET28(a)::*mcpA_C_*	This work
pGEX-6p-2::*cheA*	GST-CheA overexpression plasmid	22
pGEX-6p-2::*cheW*	GST-CheW overexpression plasmid	22
pGEX-6p-2::*cheD*	GST-CheD overexpression plasmid	22
pGB46	His_6_-C-terminal HemAT expression plasmid, pET28(a)::*hemAT_S_*	This work
pSP03	His_6_-N-terminal HemAT expression plasmid, pET28(a)::*hemAT_N_*	This work

10.1128/mBio.02177-20.9 TABLE S2 Oligonucleotides used in this study. Download Table S2, PDF file, 0.1 MB. Copyright © 2020 Tohidifar et al. 2020 Tohidifar et al. This content is distributed under the terms of the Creative Commons Attribution 4.0 International license.

Gene deletions were constructed using plasmids derived from pJSpe, which provides a CRISPR/Cas9-based, marker-free, and scarless genome-editing system for B. subtilis (68). To construct a deletion vector, a 20-bp CRISPR RNA target sequence complementary to the targeted gene sequence was designed using the CHOPCHOP online tool (69). The 5′-end-phosphorylated complementary oligonucleotides were then annealed and subcloned into BsaI restriction sites on the pJSpe plasmid using Golden Gate assembly (70). The resultant plasmid was then linearized at the SpeI restriction site and joined to two PCR fragments (∼700 to 800 bp) flanking the targeted gene using Gibson assembly (71). Prior to transformation into the B. subtilis strain, each of the pJSpe-derived deletion plasmids was linearized at the XhoI restriction site and subsequently self-ligated to create a long DNA concatemer. The concatemer was then transformed into the B. subtilis strain using the two-step Spizizen method (72). The transformation product of the B. subtilis strain and the deletion plasmid concatemer was incubated on an LB agar (LB medium and 1.5% agar) plate supplemented with 5 μg/ml kanamycin and 0.2% mannose for about 24 h at 30°C. Next, single colonies were isolated and streaked twice onto fresh drug-containing plates (described above) to ensure a clonal genotype. Positive colonies were verified using colony PCR, again streaked onto a plain LB agar plate, and incubated for an additional 24 h at 50°C to cure the deletion plasmid. Colonies with cured plasmids were unable to grow on an LB agar plate supplemented with 5 μg/ml kanamycin.

To construct chemoreceptor chimeras, two opposing primers were designed to amplify DNA regions outward from the fusion points of the chimeric gene using PCR with the pAIN750*mcpB* integration plasmid as the DNA template. Next, a second pair of primers with short overlapping regions was used to PCR amplify the desired fragment of the *mcpA* gene from pAIN750*mcpA*. Following the purification of PCR DNA products by gel extraction, the DNA fragments were assembled using Gibson assembly and transformed into E. coli. Following isolation from E. coli and sequence verification, the concatemer of the resultant integration plasmid was prepared as described above and transformed into B. subtilis OI3545, which lacks all 10 chemoreceptors. The transformation product was then incubated on an LB agar plate supplemented with 100 μg/ml spectinomycin for 15 h at 37°C. Single colonies were isolated and streaked onto a TBAB agar (TBAB and 1.5% agar) plate supplemented with 1% soluble starch. A single positive colony with the chemoreceptor expression cassette recombined to the *amyE* locus was verified using a Gram iodine solution (0.33% iodine, 0.66% potassium iodide, and 1% sodium bicarbonate). Correct colonies with a disrupted *amyE* gene were unable to form clear zones on a TBAB-starch plate.

Point mutations on the *mcpB* chemoreceptor gene were introduced using the inverse PCR method. Briefly, two opposing primers containing the desired mutations were used to PCR amplify the pAIN750*mcpB* integration plasmid. Following the purification of PCR DNA by gel extraction, the 5′ end of the DNA fragment was phosphorylated with T4 polynucleotide kinase and then blunt-end ligated using T4 DNA ligase. The ligation product was heat inactivated and transformed into E. coli. Following isolation from E. coli and sequence verification, the concatemer of the resultant integration plasmid was prepared as described above and transformed into B. subtilis OI3545 to integrate the mutant chemoreceptor expression cassette into the *amyE* locus.

Protein expression plasmids were constructed with the pET28(+) expression vector system using Gibson assembly. Briefly, DNA for the HemAT sensing domain (residues 1 to 178) was cloned in frame with a C-terminal His_6_ tag between the NcoI and HindIII restriction sites on pET28a(+). Similarly, the DNAs for the wild-type McpB, wild-type McpA, and McpB-A431S cytoplasmic regions, including the HAMP domain (residues 305 to 662 for McpB and residues 304 to 661 for McpA), were cloned in frame with a C-terminal His_6_ tag at the NcoI restriction site on pET28a(+). The DNA for the HemAT signaling domain (residues 177 to 432) was cloned in frame with an N-terminal His_6_ tag at the NheI restriction site on pET28a(+). After isolation and sequence verification, all plasmids were transformed into the E. coli BL21(DE3) strain for protein expression and purification.

### Protein expression and purification.

The CheA, CheW, and CheD proteins used in the kinase assay were expressed from glutathione *S*‐transferase (GST) fusion plasmids and purified from the E. coli BL21(DE3) strain as described previously (22, 32). GSTrap columns (5 ml; GE Healthcare) were used with an Äkta Prime fast protein liquid chromatography (FPLC) system (GE Healthcare) for purification. To purify the GST fusion proteins, cells were grown in 2 liters of LB broth with 100 μg/ml ampicillin at 37°C with shaking at 250 rpm until the optical density at 600 nm (OD_600_) reached 0.8. Expression was then induced by the addition of 1 mM IPTG (isopropyl‐β‐d‐thiogalactopyranoside), and the culture was grown for 12 h at 25°C with shaking at 250 rpm. For CheA, the culture was induced at 37°C for 4 h. Cells were then centrifuged at 8,000 × *g* for 8 min and resuspended in Tris-buffered saline (TBS) (50 mM Tris, 150 mM NaCl [pH 7.5]) supplemented with 1% Triton X-100 and 1 mM dithiothreitol (DTT) for every 1 g of cell pellet. The cells were then disrupted by sonication (5 10-s pulses). The supernatants were clarified by two rounds of centrifugation (9,000 × *g* for 15 min and 40,000 × *g* for 40 min) and loaded onto 5-ml GSTrap columns prewashed with 10 column volumes of TBS. Protein-bound columns were then washed with at least 15 volumes of TBS, and GST-tagged proteins were eluted using 10 ml glutathione elution buffer (GEB) (50 mM Tris, 5 mM glutathione [pH 8]). To remove the GST tag, the purified proteins were cleaved by PreScission protease, as specified by the supplier (Amersham Biosciences), and applied to another 5-ml GSTrap column. The flowthrough was collected and concentrated to approximately 5 ml using a cellulose ultrafiltration membrane (Millipore) in an Amicon ultrafiltration cell. Finally, the purified proteins were dialyzed in TKMD buffer (50 mM Tris, 50 mM KCl, 5 mM MgCl_2_, 0.1 mM DTT [pH 8]), and aliquots were stored at −80°C.

E. coli BL21(DE3) cells harboring the His_6_-tagged expression plasmids were grown in 2 liters of LB medium supplemented with 30 μg/ml kanamycin at 37°C with shaking at 250 rpm until the *A*_600_ reached 0.7. Expression was then induced by the addition of 1 mM IPTG, and the cultures were grown for 12 h at 25°C. Cells were harvested by centrifugation at 7,000 × *g* at 4°C for 10 min. Cells harboring HemAT_N_ were resuspended in lysis buffer (50 mM NaH_2_PO_4_, 300 mM NaCl, 10 mM imidazole [pH 8]) and sonicated (5 10-s pulses). Cell debris was removed by centrifugation at 12,000 × *g* for 1 h. The dark-red supernatant containing HemAT_N_ was loaded onto a 5-ml GE HisTrap column prewashed with NiSO_4_ and binding buffer (50 mM NaH_2_PO_4_, 300 mM NaCl, 20 mM imidazole [pH 8]). The protein-bound column was then washed with binding buffer, and proteins were eluted with elution buffer (50 mM NaH_2_PO_4_, 300 mM NaCl, 250 mM imidazole [pH 8]). The collected HemAT_N_ protein samples were concentrated using an Amicon ultrafiltration cell (Millipore) and dialyzed into dialysis buffer (50 mM Tris, 300 mM NaCl [pH 8]) at 4°C, and aliquots were stored at −80°C.

The McpB_C_, McpB_C_-A431S, McpA_C_, and HemAT_S_ proteins were purified under denaturing conditions. Briefly, cells were induced and grown as described above. Cells were then resuspended in buffer B (8 M urea, 0.1 M NaH_2_PO_4_, 0.01 M Tris [pH 8]) with 1% Triton X-100 and 1 mM DTT for every 1 g of cell pellet and incubated at room temperature for 1 h. The cell suspension was clarified by centrifugation at 40,000 × *g* for 1 h. The cell lysates were loaded onto a 5-ml GE Hi-Trap chelating column charged with 0.1 M NiSO_4_ and washed with buffer B and buffer C (buffer B at pH 6.3). The fusion proteins were eluted from the column with 25 ml elution buffer E (buffer B at pH 4.5). Proteins were refolded by dialysis in phosphate-buffered saline (PBS) (10 mM Na_2_HPO_4_, 1.8 mM KH_2_PO_4_, 137 mM NaCl, 2.7 mM KCl [pH 7.4]) at 4°C, and aliquots were stored at −80°C. The proper folding of purified proteins was verified by circular dichroism (CD) spectroscopy. The concentrations of all purified proteins were quantified by a Pierce bicinchoninic acid (BCA) protein assay kit. SDS-PAGE images of the purified recombinant chemoreceptor proteins are provided in Data Set S1.

10.1128/mBio.02177-20.10 DATA SET S1 Raw data for all experiments, except STD-NMR, and SDS-PAGE images of purified recombinant chemoreceptor proteins reported in the manuscript. Download Data Set S1, XLSX file, 0.3 MB. Copyright © 2020 Tohidifar et al. 2020 Tohidifar et al. This content is distributed under the terms of the Creative Commons Attribution 4.0 International license.

### Capillary assay for chemotaxis.

The capillary assay was performed as described previously (73). Briefly, cells were grown for 16 h at 30°C on TBAB plates. The cells were then scraped from the plates and resuspended to an OD_600_ of 0.03 in 5 ml CAMM supplemented with 50 μg/ml histidine, 50 μg/ml methionine, 50 μg/ml tryptophan, 20 mM sorbitol, and 2% TB. The cultures were grown to an OD_600_ of 0.4 to 0.45 at 37°C with shaking at 250 rpm. At this point, 50 μl of GL solution (5% [vol/vol] glycerol and 0.5 M sodium lactate) was added, and cells were incubated for another 15 min (at 37°C with shaking at 250 rpm). The cells were then washed twice with chemotaxis buffer and incubated for an additional 25 min (at 37°C with shaking at 250 rpm) to ensure that the cells were motile. The cells were then diluted to an OD_600_ of 0.001 in chemotaxis buffer and aliquoted into 0.3-ml ponds on a slide warmer at 37°C, and closed-end capillary tubes filled with alcohol solutions or an asparagine solution (3.16 μM) prepared in the same chemotaxis buffer were inserted. After 30 min, cells in the capillaries were harvested, transferred to 3 ml of top agar (1% tryptone, 0.8% NaCl, 0.8% agar, and 0.5 mM EDTA), and plated onto TB agar (TB and 1.5% agar) plates. These plates were incubated for 16 h at 37°C, and colonies were counted. Experiments were performed in triplicate each day and repeated on three different days.

### Cell growth.

Cell density was measured as the optical absorbance at 600 nm. Briefly, B. subtilis 168 was first grown for 16 h at 30°C on a TBAB plate. For growth experiments in minimal medium, the cells were first scraped from the TBAB plate and then resuspended to an OD_600_ of 0.03 in 50 ml CAMM supplemented with 50 μg/ml tryptophan and 5 g/liter glucose and grown at 37°C with shaking at 250 rpm. At an OD_600_ of 0.8, the cells were diluted 1:20 (vol/vol) into 50 ml CAMM containing 50 μg/ml tryptophan supplemented with 0.01 M ethanol, 0.1 M ethanol, or 5 g/liter glucose (positive control) and grown for 24 h at 37°C with shaking at 250 rpm. For growth experiments in rich medium, cell cultures starting at an OD_600_ of 0.03 were grown to an OD_600_ of 0.4 at 37°C with shaking at 250 rpm in 50 ml LB medium. At this point, cell cultures were supplemented with 0.01 M, 0.1 M, or 1.0 M ethanol and grown for another 5 h at 37°C with shaking at 250 rpm. All growth experiments were performed in triplicate.

### Ethanol utilization experiments.

Ethanol concentrations were measured using a Shimadzu high-performance liquid chromatography system equipped with a RID-10A refractive index detector, an Aminex HPX-87H carbohydrate analysis column (Bio-Rad Laboratories), and a cation H micro-guard cartridge (Bio-Rad Laboratories). The column and guard cartridge were kept at 65°C, and 0.5 mM H_2_SO_4_ was used a mobile phase at a constant flow rate of 0.6 ml/min. Prior to measurements, cells in culture samples were pelleted, and the resulting supernatant was passed through a 0.22-μm polyethersulfone syringe filter. Peaks were identified and quantified by retention time comparison to the standards.

### Alcohol dehydrogenase activity measurement.

B. subtilis OI1085 was first grown for 16 h at 30°C on a TBAB plate. For aerobic growth, the cells were then scraped from the TBAB plate; resuspended to an OD_600_ of 0.03 in 5 ml CAMM supplemented with 50 μg/ml histidine, 50 μg/ml methionine, 50 μg/ml tryptophan, 20 mM sorbitol, and 2% TB; and grown at 37°C with vigorous shaking at 250 rpm. For anaerobic growth, however, cells were cultured starting at an OD_600_ of 0.03 in a sealed bottle filled to the top without agitation in CAMM supplemented with 1% glucose and a mixture of all 20 amino acids at 50 μg/ml (29). For E. coli cultures, the cells (MG1655) were grown in M9 medium supplemented with 0.4% glucose at 37°C in sealed bottles filled to the top without agitation for anaerobic growth and in flasks with shaking at 250 rpm for aerobic growth (31). All cell cultures were grown to stationary phase prior to sonication (7 10-s pulses), and soluble cell extracts were obtained by centrifugation (7,000 × *g* at 4°C for 10 min). Alcohol dehydrogenase enzyme assays were performed as described previously (74). Briefly, the assay reaction mixtures were prepared with 22 mM sodium pyrophosphate (pH 8.8), 0.3 mM sodium phosphate, 7.5 mM β-NAD, 0.003% (wt/vol) bovine serum albumin, 1.6% (vol/vol) of the desired cell lysate, and 3.2% (vol/vol) ethanol in a 200-μl reaction volume. Next, the reduction of NAD^+^ to NADH was recorded at 340 nm using a Shimadzu UV-1800 spectrophotometer. One unit of alcohol dehydrogenase activity is defined as the amount of enzyme that converts 1 μmol of ethanol to acetaldehyde per min at pH 8.8 at 25°C.

### Antimicrobial diffusion assay.

The antifungal activities of the B. subtilis strains were assayed using the disc diffusion method as described previously (75). Briefly, S. cerevisiae CEN.PK113-7D was grown in YPD rich medium for 24 h at 30°C with shaking at 200 rpm. A total of 0.1% (vol/vol) of the yeast culture was mixed with YPD top agar (YPD medium with 0.8% agar) and spread on top of a YPD plate (YPD medium with 2% agar). Once the top yeast layer was solidified, 10-mm filter paper (Whatman filter paper, grade 1) discs loaded with supernatants from B. subtilis strains grown overnight in LB medium at 37°C were placed on top of the yeast layer. As negative controls, separate discs were loaded with LB broth and water. The plate was incubated at 30°C for another 24 h and then imaged. A zone of inhibition around the discs indicated antifungal activity.

### Preparation of bacterial membranes.

Cells were grown for 16 h at 30°C on TBAB plates. The cells were then scraped from the plates and resuspended to an OD_600_ of 0.03 in 50 ml CAMM supplemented with 50 μg/ml histidine, 50 μg/ml methionine, 50 μg/ml tryptophan, 20 mM sorbitol, and 2% TB. The cells were grown at 37°C with aeration until they reached mid-exponential phase. The cells were then diluted 1:10 (vol/vol) into 50 ml CAMM and grown until mid-exponential phase. The cells were again diluted to an OD_600_ of 0.01 in 50 ml medium and grown until mid-exponential phase. Finally, the cultures were diluted 1:10 (vol/vol) into multiple flasks containing 50 ml medium and grown with shaking at 37°C until an OD_600_ of 0.6 was reached. The cells were then harvested by centrifugation at 9,900 × *g* for 15 min and washed 3 times with 1 M KCl to remove extracellular proteases. Cells were resuspended in sonication buffer plus (10 mM potassium phosphate [pH 7], 10 mM MgCl_2_, 1 mM EDTA, 0.3 mM DTT, 20 mM KCl, 1 mM glutamate, 2 mM phenylmethanesulfonyl fluoride, and 20% glycerol). EDTA and phenylmethanesulfonyl fluoride were added as protease inhibitors. Cells were sonicated, and the cell debris was removed by centrifugation at 17,600 × *g* at 4°C for 15 min. Bacterial membranes were removed by centrifugation at 120,000 × *g* for 2 h at 4°C in a Beckman 70 Ti rotor. Pelleted membranes were resuspended in MT buffer (10 mM potassium phosphate [pH 7], 1 mM MgCl_2_, 0.1 mM EDTA, and 1 mM 2-mercaptoethanol) and homogenized using a glass-Teflon homogenizer, followed by another centrifugation step at 120,000 × *g* for 2 h at 4°C. This step was repeated once more. Finally, the membranes were homogenized in MT buffer at a concentration of 32 mg/ml and stored in small aliquots at −80°C.

### *In vitro* assay for receptor-coupled kinase activity.

Reaction mixtures consisted of purified B. subtilis membranes expressing McpB or HemAT as the sole chemoreceptor and purified CheW, CheA, and CheD prepared in buffer (50 mM Tris, 50 mM KCl, 5 mM MgCl_2_ [pH 7.5]) at the following concentrations: 6 μM chemoreceptor, 2 μM CheW, 2 μM CheA kinase, and 2 μM CheD. Ethanol was then added to the mixture at different final concentrations in a 20-μl reaction volume. As a negative control, only buffer was added. Reaction mixtures were then preincubated at 23°C for 1 h to permit the formation of the chemoreceptor-kinase complex. CheA autophosphorylation was initiated by the addition of [γ-^32^P]ATP (4,000 to 8,000 cpm/pmol) to a final concentration of 0.1 mM. Five-microliter aliquots were quenched at 15 s by mixing the reaction mixtures with 15 μl of 2× Laemmli sample buffer containing 25 mM EDTA at room temperature, essentially fixing the level of phosphor-CheA. Initial phosphor-CheA formation rates were analyzed using 12% SDS-PAGE gels. Gels were dried immediately after electrophoresis, and phosphor-CheA was quantified by phosphorimaging (Molecular Dynamics) and ImageJ (76).

### Circular dichroism spectroscopy.

Far-UV CD spectra were measured on a Jasco J-720 spectropolarimeter (Japan Spectroscopic Co., Inc., Tokyo, Japan) with a cuvette with a path length of 0.1 cm. Prior to measurements, protein samples were dialyzed into 10 mM sodium phosphate buffer (pH 8) and diluted to 2.5 μM. Spectral measurements were carried out in triplicate using a scanning rate of 50 nm/min and a 0.1-nm step size with 5 accumulations per sample. A buffer-only control sample was used for baseline correction, and curves were smoothed according to the Savitzky-Golay algorithm (77). Structural analysis was done using BeStSel (78).

### UV-visible spectral measurements.

All UV spectral measurements were performed on a Shimadzu UV-1800 spectrophotometer. The UV spectra of the oxygenated sensing domain of the HemAT (HemAT_N_) protein were measured under aerobic conditions. To measure the UV spectra of HemAT_N_ in the presence of ethanol, protein samples were first deoxygenated by adding a few grains of sodium dithionite in a glove box. Sodium dithionite-reduced protein samples were then titrated with different doses of ethanol in sealed quartz cuvettes, and the UV spectra (200 nm to 600 nm) of these samples were immediately recorded in the spectrophotometer.

### Saturation transfer difference nuclear magnetic resonance spectroscopy.

All nuclear magnetic resonance (NMR) spectroscopy measurements were performed on a Varian VNMRS instrument at 750 MHz with a 5-mm Varian HCN probe at 298 K without sample spinning. Prior to measurements, protein samples were buffer exchanged into PBS (50 mM KH_2_PO_4_, 20 mM NaCl [pH 7.4]) in D_2_O using Micro Bio-Spin columns with Bio-Gel P-6 (Bio-Rad Laboratories, Hercules, CA, USA). To avoid aggregation, HemAT_N_ protein was buffer exchanged into modified PBS (50 mM KH_2_PO_4_, 300 mM NaCl [pH 8.0]) containing 10% D_2_O. Protein samples at 50 μM were then mixed with the alcohol (final concentration of 3 mM) in a 500-μl solution. ^1^H spectra were obtained from 32 scans with a 90° pulse and a 2-s relaxation delay. In saturation transfer difference NMR (STD-NMR) experiments, the protein samples were selectively saturated at 2.15 ppm with a train of Gaussian pulses of a 50-ms duration with a 0.1-ms delay and a 5-s relaxation delay for a total saturation time of 3 s and 2,048 scans. Off-resonance irradiation was applied at 30 ppm. A trim pulse of 50 ms was used to reduce protein background. In the case of HemAT_N_, the protein sample was saturated at 7.06 ppm, and 256 scans were used to obtain spectra. All STD spectra were obtained by internal subtraction via phase cycling after a block size of 8 to reduce artifacts resulting from temperature variation and magnet instability. Control experiments were performed on samples containing only the alcohol without protein. All areas were calculated using MNova V14.1 (by Mestrelab Chemistry Solutions) in stacked mode.

### Structural analysis.

Domains of the McpB, McpA, TlpA, and TlpB chemoreceptors from B. subtilis were predicted using the phmmer search engine on the HMMER Web server using the UniProt reference proteome database with default sequence E value thresholds (79). The amino acid sequences of the cytoplasmic signaling domains were then manually obtained based on previous large-scale alignment results (10). To identify the three structural subdomains of the cytoplasmic signaling domain, the sequences were then aligned with the amino acid sequences of the corresponding domains from the Tar, Tsr, Trg, and Tap chemoreceptors of E. coli using MUSCLE (80) with default parameter values. Pairwise amino acid sequence alignments between the protein pairs (McpA-McpB, McpA-HemAT, and HemAT-YfmS) for chimeric receptor analysis were performed using EMBOSS Water (81). A homology model of the cytoplasmic signaling domain of the McpB dimer (residues 352 to 662) was constructed in Modeller (v-9.23) (82) using the Thermotoga maritima Tm113 chemoreceptor (PDB accession number 2CH7) as the template (83). Side chain conformations were refined using SCWRL4 (84), and the entire structural model was subsequently refined using the YASARA energy minimization server (85). The resulting Ramachandran plots were verified using Procheck (86). The crystal structure of the HemAT sensing domain from B. subtilis (PDB accession number 1OR6) (40) was used for visualization. Visualization of all structures was accomplished using the VMD software package (v-1.9.3) (87).

### Receptor-ligand *in silico* docking experiment.

The putative binding sites for ethanol were determined using Autodock (v-4.0) (88). Briefly, hydrogen atoms were first added to the McpB cytoplasmic signaling domain dimer model, and the number of torsional degrees of freedom for ethanol was set at 1. Autogrid was then used to adjust the position of grid boxes (60 by 60 by 60 points with 0.375-Å spacing for each box) on the ethanol-sensing region (residues 390 to 435). Finally, the Lamarckian genetic algorithm was employed to obtain the best docking site configurations.

### Molecular dynamics simulations.

All-atom molecular dynamics simulations were conducted using NAMD 2.13 (89) and the CHARMM36 force field (90). Simulations were carried out using the NPT ensemble (pressure of 1 atm and temperature of 310 K) with values for general simulation parameters as previously described (91). The McpB cytoplasmic dimer model was solvated with TIP3P (transferable intermolecular potential with 3 points) water and 150 mM NaCl using VMD (87), and 165 ethanol molecules (0.316 M) were randomly placed within the simulation box using the gmx insert-molecules tool. A copy of the system that included the A431S mutation was created, and both the wild-type and mutant McpB-ethanol systems were subjected to conjugant gradient energy minimization (2,000 steps), followed by a 10-ns equilibration simulation with protein backbone restraints and 3 600-ns unrestrained production simulations.

### Molecular dynamics simulation analysis.

Density maps representing the average ethanol occupancy were computed using the VolMap plug-in in VMD with default settings and averaging over each production simulation for the wild-type and A431S mutant McpB-ethanol systems. To highlight unique binding sites between the two maps, a difference map was computed by subtracting the A431S map from the wild-type map using VMD’s volutil plug-in and removing smaller volumes resulting from slight irregularities in overlapping sites using the “hide dust” feature in UCSF Chimera. All densities are visualized at an isovalue of 0.03 except for the difference map, which used an isovalue of 0.015. Protein-ethanol coordination was computed by measuring the minimum distance between nonhydrogen atoms in each residue and the nearest ethanol molecule; if this distance was less than 4 Å, the pair was considered to be in contact. Average coordination values were computed for each residue in the wild-type and A431S mutant McpB-ethanol systems by averaging over all three production simulations at 200-ps intervals. Percent changes were obtained by subtracting the values obtained in the latter from those obtained in the former. Knobs-in-holes packing within the McpB cytoplasmic signaling domain was analyzed using the program SOCKET (92) with a packing cutoff of 7.8 Å (93). For each production simulation, knobs-in-holes packing was assessed at 2-ns intervals over the course of the trajectory, not including the first 100 ns to allow for packing changes resulting from equilibration or the A431S mutation. The occupancy of a particular knob-in-hole interaction over a given simulation was taken as the number of intervals in which it was identified by SOCKET divided by the total number of intervals analyzed in the simulation. The reported knobs-in-holes occupancies were averaged over both McpB monomers and all three production simulations for each McpB-ethanol system; error bars denote 1 standard deviation from the mean.

### Simulation of ethanol diffusion in the capillary assay.

The spatiotemporal evolution of ethanol (*C*) in the capillary assay was modeled using Fick’s second-law equation with Neumann (no-flux) boundary conditions shown in the following equation: ∂*C*/∂*t* = *D*Δ*C*. The initial ethanol concentrations were set to 50 mM in the capillary and 0 mM in the pond. The ethanol diffusion coefficient (*D*) was assumed to be 1.23 × 10^−3^ mm^2^/s (94). The above-described partial differential equation was solved using the finite-element method with the help of FEniCS (v-2019.1.0), an open-source computing platform (95). Briefly, the computation domain consists of the capillary and the proximal region near the mouth of the capillary in the pond. The capillary was modeled as a 10-mm-long cylinder with a diameter of 0.2 mm attached to an 8-mm-long cylinder with a diameter of 4 mm. Gmsh (v-4.5.2) (96) was used to generate the three-dimensional finite-element mesh, and the XML file of the resulting mesh was produced using the meshio-convert tool available from FEniCS. The implicit Euler method was employed for time integration with a step size of Δ*t* = 1 s. A custom Python script was generated for solving the finite-element problem.

### Data availability.

Raw data for all experiments except STD-NMR are provided in Data Set S1 in the supplemental material. Raw data for the STD-NMR experiments are available upon request. The Python script for diffusion simulation is provided at https://github.com/paymantohidifar/alcoholtaxis.
