# Supplementary figures and images for: The Unconventional Cytoplasmic Sensing Mechanism for Ethanol Chemotaxis in Bacillus subtilis
Source: mBio. 2020 Oct 6;11(5):e02177-20. doi: 10.1128/mBio.02177-20 (PMC7542364; doi:10.1128/mBio.02177-20)

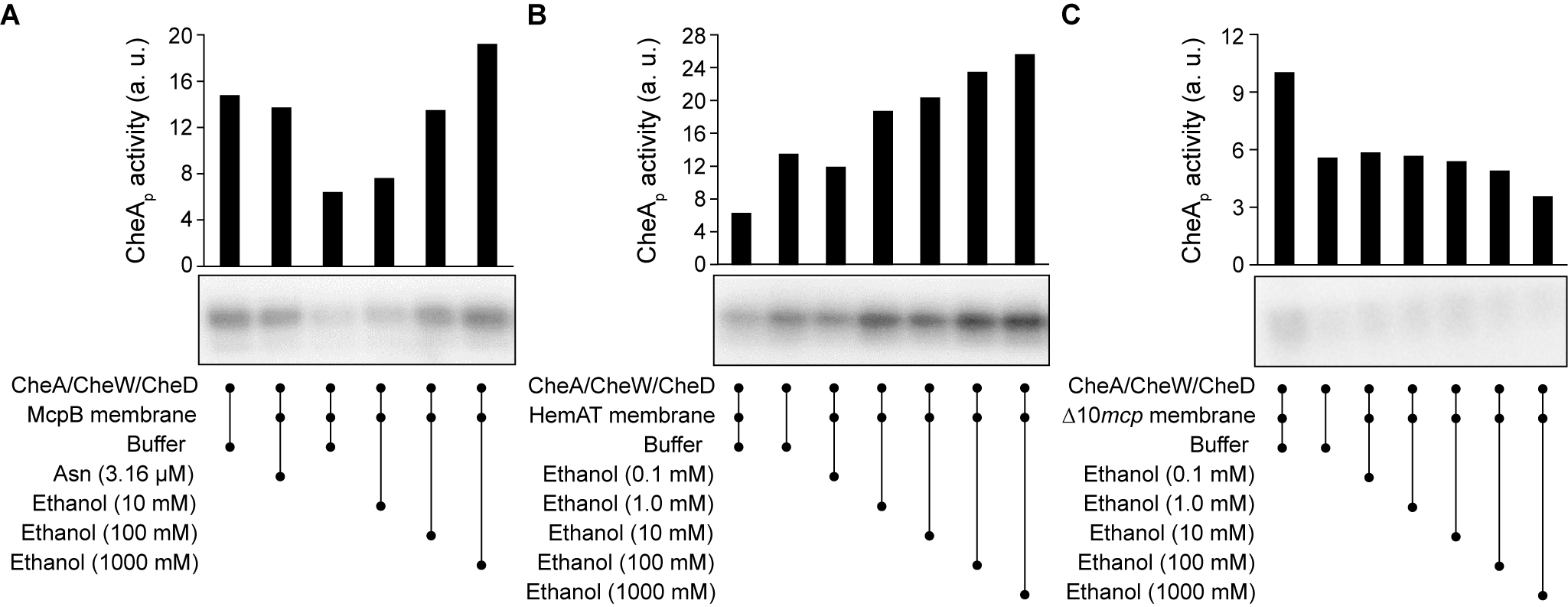

Supplement: FIG S1 [file mBio.02177-20-sf001.tif]

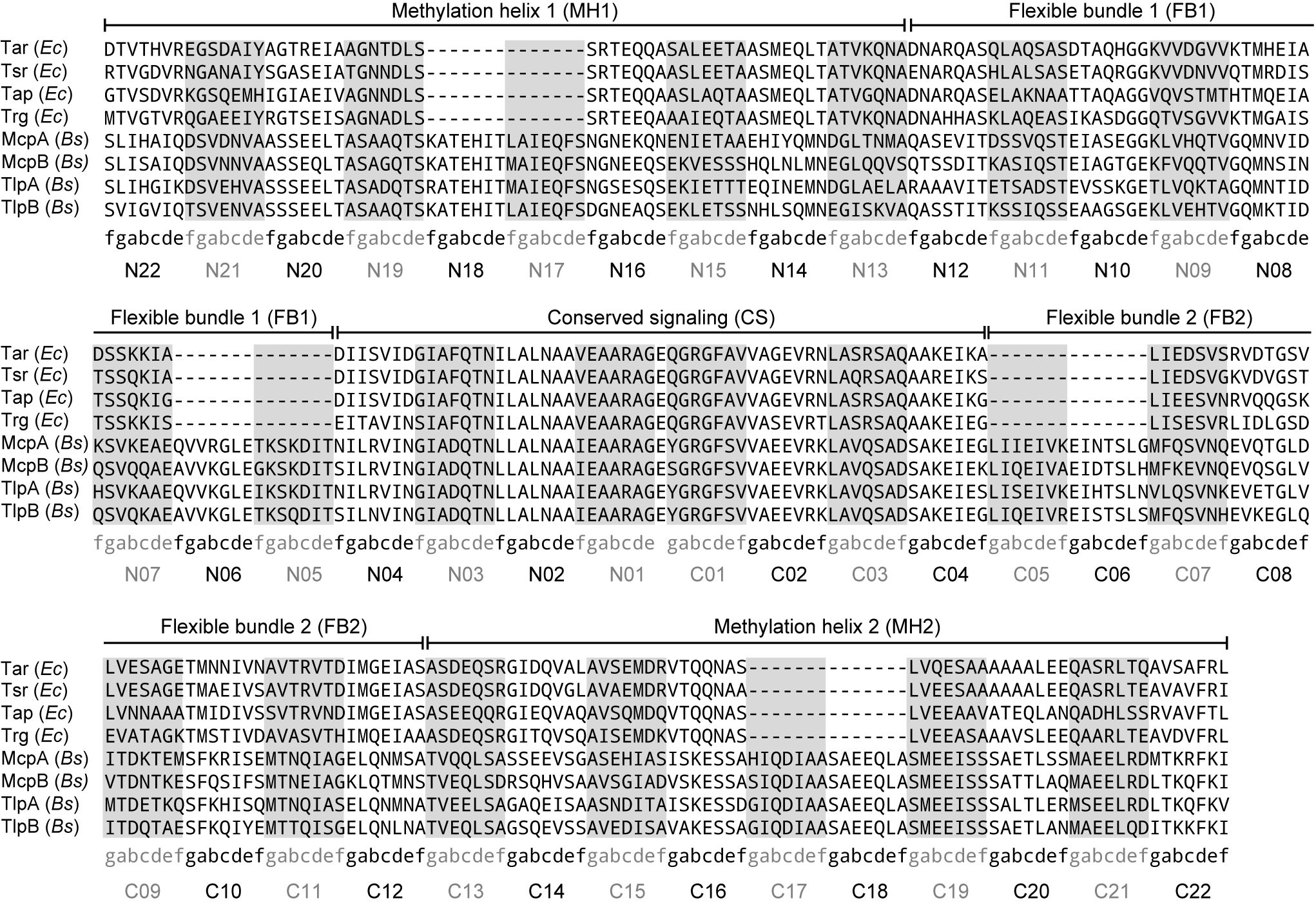

Supplement: FIG S2 [file mBio.02177-20-sf002.tif]

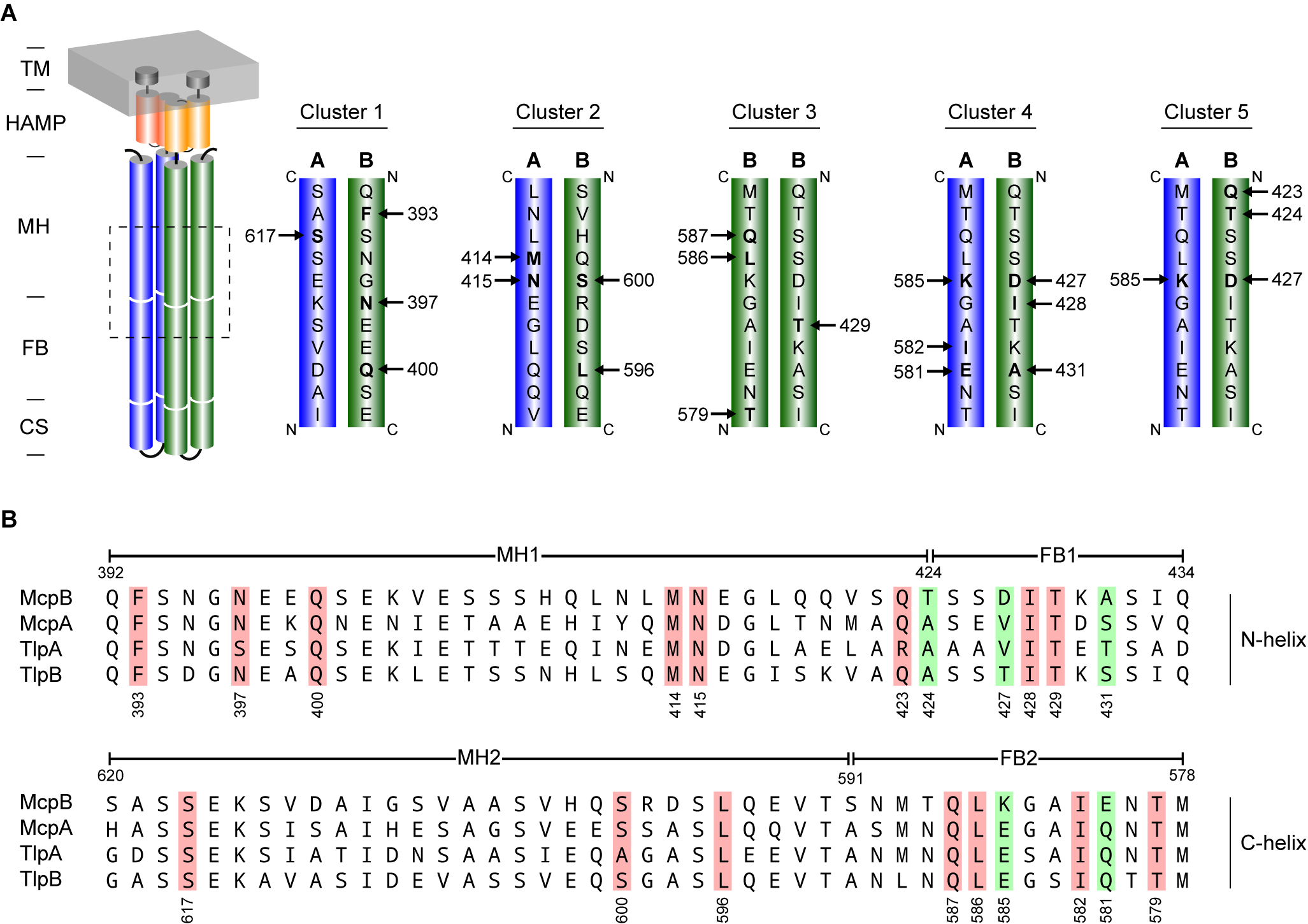

Supplement: FIG S3 [file mBio.02177-20-sf003.tif]

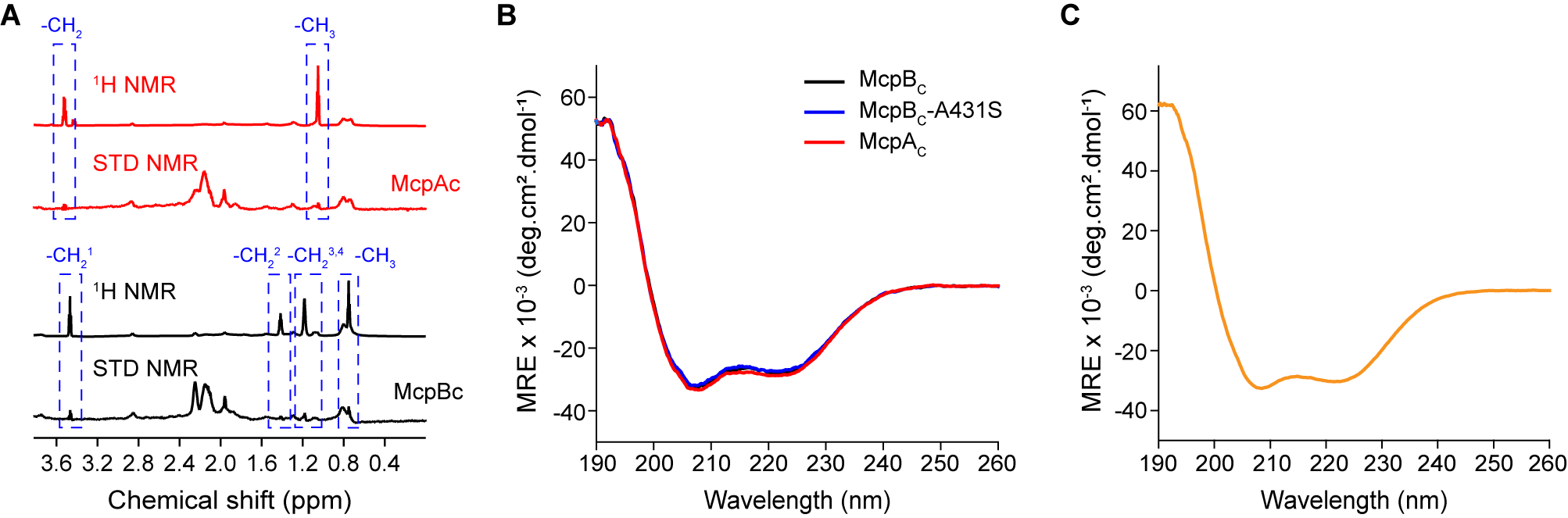

Supplement: FIG S4 [file mBio.02177-20-sf004.tif]

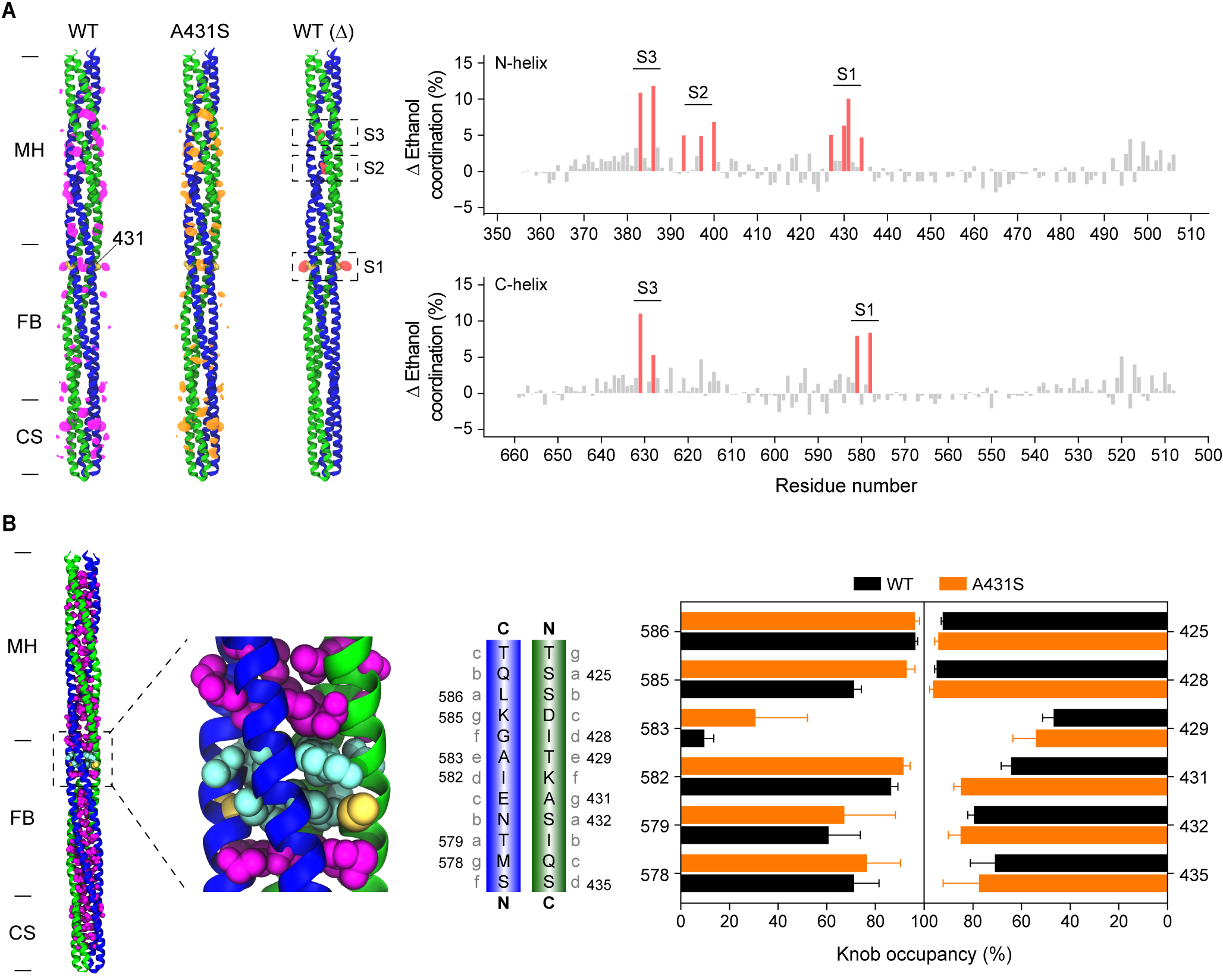

Supplement: FIG S5 [file mBio.02177-20-sf005.tif]

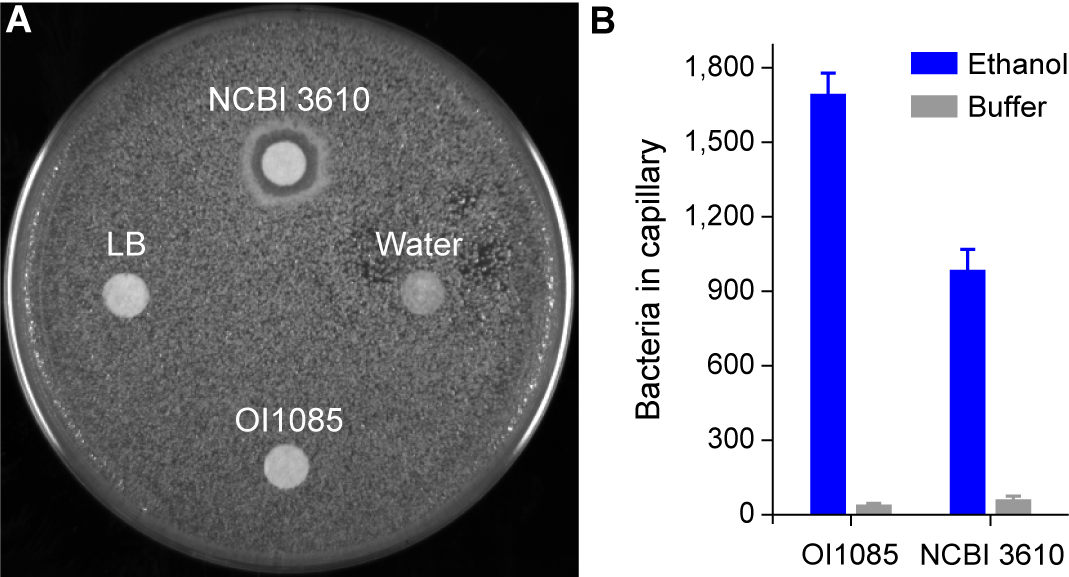

Supplement: FIG S6 [file mBio.02177-20-sf006.tif]

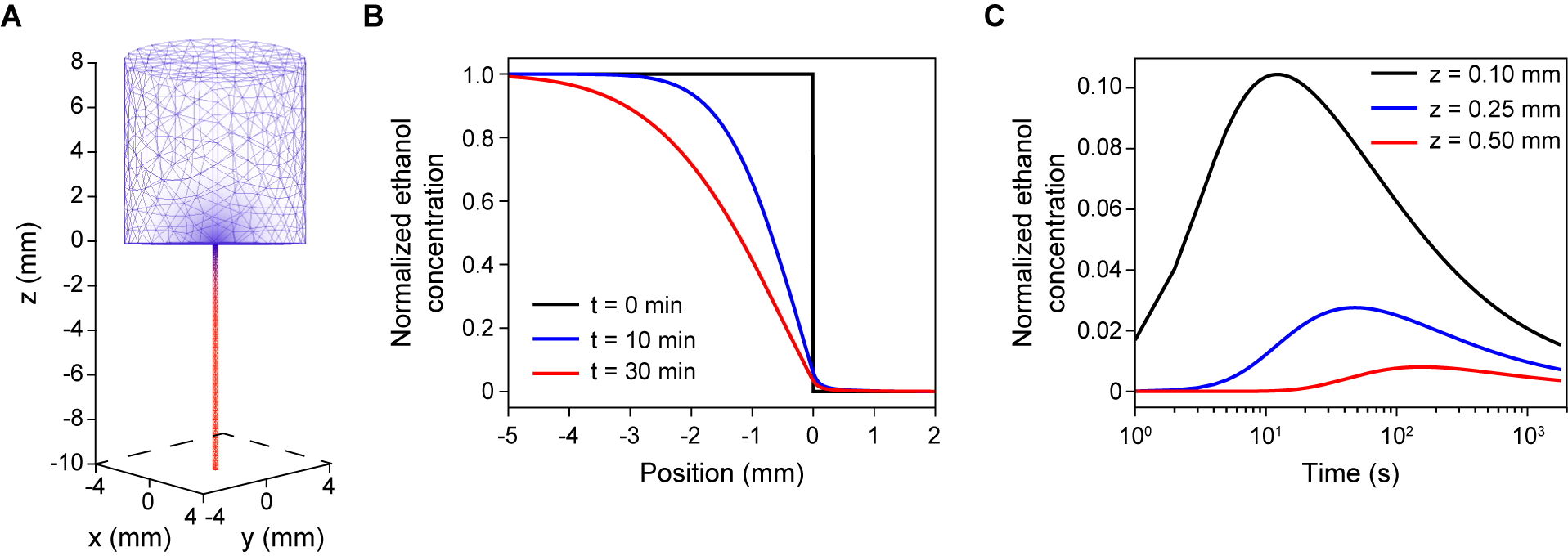

Supplement: FIG S7 [file mBio.02177-20-sf007.tif]
