# Supplementary material for: The Unconventional Cytoplasmic Sensing Mechanism for Ethanol Chemotaxis in Bacillus subtilis
Source: mBio. 2020 Oct 6;11(5):e02177-20. doi: 10.1128/mBio.02177-20 (PMC7542364; doi:10.1128/mBio.02177-20)
Supplement: TABLE S1 [file mBio.02177-20-st001.pdf]

Table S1. Putative ethanol-binding sites within the McpB ethanol-sensing region predicted by *in silico* docking experiments.

Cluster	Predicted interacting residues		Score (kcal/mol)
	Chain A	Chain B	
1	Phe^{393}, Asn^{397}, Gln^{400}	Asn^{397}, Gln^{400}, Ser^{617}	-2.71
2	Met^{414}	Met^{414}, Asn^{415}, Leu^{596}, Ser^{600}	-2.1
3	-	Thr^{429}, Thr^{579}, Leu^{586}, Gln^{587}	-1.97
4	Glu^{581}, Ile^{582}, Lys^{585}	Asp^{427}, Ile^{428}, Ala^{431}	-1.84
5	Lys^{585}	Gln^{423}, Asp^{427}, Thr^{424}	-1.78
