# Supplementary material for: The Unconventional Cytoplasmic Sensing Mechanism for Ethanol Chemotaxis in Bacillus subtilis
Source: mBio. 2020 Oct 6;11(5):e02177-20. doi: 10.1128/mBio.02177-20 (PMC7542364; doi:10.1128/mBio.02177-20)
Supplement: TABLE S2 [file mBio.02177-20-st002.pdf]

Table S2. Oligonucleotides used in this study.

Name	Sequence	Purpose
PT200F	5'ACCAGCATAGTAAGAATAGCCATGTCTACACATTATGAAAC	PCR of homology templates for *hemAT* gene deletion
PT200R	5'GTTTTTGATGGTACAATGATCCCCCTTGG	
PT201F	5'GATCATTGTACCATCAAAAACCGGTCTG	
PT201R	5'CCGATGTCCAGTAAGACAGTCTGGATCGCTACAC	
PT116F	5'CCGATCGACTTTTCTTCAAC	Target sequence for *hemAT* gene deletion
PT116R	5'AAACGTTGAAGAAAAGTCGATCGG	
PT558F	5'CACAATCATTCCTGTTGTCAG	Long PCR for construction of pPT200
PT558R	5'AAACCGAAAAACAGCGCTATC	
PT559F	5'AGCGCTGTTTTTCGGTTTTTATTCAATTTTAAATCGTTTGGTCATATC	PCR of insert for construction of pPT200
PT559R	5'ACAACAGGAATGATTGTGTTGGCCGCAGCTATTATC	
PT567F	5'CTGAATAGCGCTGATCAGAG	Long PCR for construction of pPT205
PT567R	5'AAACCGAAAAACAGCGCTATC	
PT568F	5'AGCGCTGTTTTTCGGTTTTTATTCAATTTTAAATCGTTTGGTCATATC	PCR of insert for construction of pPT205
PT568R	5'TGATCAGCGCTATTCAGGATTCGGTAGACAATGTTGC	
GB050F	5'TGCGGTAAGCTGTTCGGAAG	Long PCR for construction of pGB42
GB050R	5'AAACCGAAAAACAGCGCTATC	
GB051F	5'ATAGCGCTGTTTTTCGGTTTTTATTCAATTTTAAATCGTTTGGTCATATCCCG	PCR of insert for construction of pGB42
GB051R	5'CTTCCGAACAGCTTACCGCATCAGCTGCGCAAACGAGC	
GB052F	5'GTTTCCGTTTGAGAACTGC	Long PCR for construction of pGB43
GB050R	5'AAACCGAAAAACAGCGCTATC	
GB051F	5'ATAGCGCTGTTTTTCGGTTTTTATTCAATTTTAAATCGTTTGGTC	PCR of insert for construction of pGB43
GB053R	5'AGCAGTTCTCAAACGGAAACGAAAAACAAAACGAAAACATTG	
GB034F	5'GATAGATGCCTTTGTAATATCTGAC	Long PCR for construction of pGB34
GB034R	5'AAACCGAAAAACAGCGCTATC	
GB035F	5'TAGCGCTGTTTTTCGGTTTTTATTCAATTTTAAATCGTTTGGTCATATC	PCR of insert for construction of pGB34
GB035R	5'ATATTACAAAGGCATCTATCCAATCGACAGAAATTGCAAGTG	

Table S2. Oligonucleotides used in this study (continued).

Name	Sequence	Purpose
GB077F	5’TTGTGAGACTTGCTGGAG	Long PCR for construction of pGB64
GB073R	5’AAACCGAAAAACAGCGCTATC	
GB076F	5’ATAGCGCTGTTTTTCGGTTTTTATTCAATTTTAAATCGTTTGGTC	PCR of insert for construction of pGB64
GB078R	5’GACTCCAGCAAGTCTCACAAGCTTCTGAAGTGATCACTG	
GB079F	5’GATTGGATAGATGACTTTGTAATATCTGAC	Construction of pGB65
GB079R	5’AACGGAAATCGCCGGAACG	
GB101F	5’ATATCTGACGATGCTTGTGAGACTTG	Construction of pGB83
GB101R	5’TACAAAGGCATCTATCCAATCAACG	
GB100F	5’CCTTTGTAATAGTTGACGATGTTTG	Construction of pGB82
GB100R	5’CATCTATCCAATCAACGGAAATC	
GB081F	5’TGCCCGCGATTTGGTTTGTCATG	Construction of pGB67
GB081R	5’AGCTGCAGACGATGAATTCCACC	
GB098F	5’ATCGTCTGCAGCTCGCCCGCG	
GB098R	5’GAATTCCACCGTCGAGCAGCTGTCAG	Construction of pGB79
GB036F	5’CAAAATGCTTGTGATGTCTTTTG	
GB034R	5’AAACCGAAAAACAGCGCTATC	Long PCR for construction of pGB35
GB035F	5’ATAGCGCTGTTTTTCGGTTTTTATTCAATTTTAAATCGTTTGGTCATATC	
GB037R	5’AAGACATCACAAGCATTTTGCGTGTGATCAACGGCATC	PCR of insert for construction of pGB35
PT279F	5’GTCACTTGCTCCTTCAGG	
PT279R	5’GACTCGGTGAACAATGTG	Long PCR for construction of pPT086
PT280F	5’CATTGTTCACCGAGTCCTGGATGGCGTGTATGAG	
PT280R	5’TGAAGGAGCAAGTGACATGAAAAAAATACTCCAACTCATAAAAC	PCR of insert for construction of pPT086
GB024F	5’GCCGCGCGGCAGCCATATGGCCCTTGAAGCGTTTCAAAGC	
GB024R	5’TTTGCTGTCCACCAGTCATGTTATTCTTCTGTCAGGATGAC	Construction of pGB046
PT618F	5’CTTTAAGAAGGAGATATACATGTCGATTACAAAACCGTTAAAAC	Construction of pPT262, pGB78
PT618R	5’TCGAGTGCGGCCGCAAGCTCGATTTTAAATTGTTTCGTC	
SP014F	5’GGTGCTCGAGTGCGGCCGCATACGCTTCAAGGACAAGC	Construction of pSP03
SP014R	5’ACTTTAAGAAGGAGATATACATGTTATTTAAAAAAGACAGAAAACAAG	
GB058F	5’CTTTAAGAAGGAGATATACATGTCGATTACAACACCGCTG	Construction of pGB053
GB058R	5’TCGAGTGCGGCCGCA*AGCT*CAATTTTAAATCGTTTGGTCATATCC	

Table S2. Oligonucleotides used in this study (continued).

Name	Sequence	Purpose
GB116F	5'TTCGCTTTGCTTCTCGTTTCCG	Construction of pGB094
GB116R	5'AAGGTTGAATCCAGCTCTCATC	
